# Small glycomimetic antagonists of the cytomegalovirus glycoprotein UL141 prevent binding to TRAIL death receptor

**DOI:** 10.1016/j.jbc.2025.108490

**Published:** 2025-04-10

**Authors:** Ivana Nemčovičová, Juraj Kóňa, Monika Poláková, Tomáš Klunda, Andrej Bitala, Mário Benko, Simona Lenhartová, Marek Nemčovič

**Affiliations:** 1 Biomedical Research Center (BMC), Slovak Academy of Sciences, Bratislava, Slovakia; 2 Institute of Chemistry, Slovak Academy of Sciences, Bratislava, Slovakia; 3 Medical Vision, o. z., Bratislava, Slovakia

**Keywords:** HCMV UL141, viral protein antagonist, glycomimetic, cell surface TNF receptor, immune evasion, *in silico* analysis, protein-protein interaction, molecular docking, FMO pair interaction energy, iminosugar

## Abstract

Human cytomegalovirus (HCMV) UL141 inhibits immune recognition of virally infected cells by natural killer cells and cytotoxic T cells through modulation of cellular receptors (*e.g.*, TRAIL-R2/-R1, CD155, CD112). Recent findings suggest that UL141 is also a critical component of the HCMV virion, further emphasizing its significance. In this study, we aimed to develop a small synthetic compound as a UL141 antagonist. Building on our crystal structure analysis, we designed compounds to specifically bind viral UL141, thereby blocking its interaction with target receptors thus inhibiting its immunoevasive functions. We evaluated a small library of synthesized compounds composed of diverse saccharide units conjugated with nonsaccharide moieties, such as nonionic glycolipids, pyrrolidines, and “click” conjugates. An ELISA-like TMB-binding assay, coupled with dynabeads coating, was employed to assess the ability of these compounds to inhibit TRAIL-R2 binding *in vitro*. The most promising compounds capable of inhibiting complex formation were further analyzed using surface plasmon resonance. Compound 18 exhibited the strongest binding affinity to UL141, with KD of 2.93 μM. Molecular docking studies identified specific binding sites on UL141, and the fragmented molecular orbital method was applied to evaluate interaction energy patterns between the antagonist and the UL141 protein. Mutational analysis was conducted to validate the identified binding sites on UL141. Additionally, cellular cytotoxicity assays were performed to confirm the nontoxic properties of these compounds. Collectively, our findings suggest that synthetic glycomimetics represent promising candidates for targeting the viral glycoprotein HCMV UL141, thereby disrupting TRAIL death receptor signaling, thus mitigating viral activity.

Human cytomegalovirus (HCMV) establishes lifelong persistence despite robust humoral and cellular immune responses. This virus poses a significant threat to immunocompromised individuals, including transplant recipients, individuals with HIV, and those affected by congenital infection. HCMV infection is associated with lifelong health complications, contributing to substantial global healthcare costs, estimated as billions in annual costs in the United States alone (1). A major challenge in vaccine design is the incomplete understanding of the diverse protein complexes the virus uses to infect cells. Consequently, the development of a vaccine against HCMV, particularly for the prevention of congenital disease, remains a top priority (1, 2, 3). Despite this urgency, no vaccine has yet been licensed. Current treatment options rely on antiviral agents, including inhibitors of HCMV DNA polymerase, the terminase complex, or viral protein kinases. However, these therapies are limited by severe toxic side effects and the emergence of drug-resistant viral strains (4). Furthermore, existing antiviral treatments target only the lytic phase of HCMV, leaving latent infections untreatable and viral reservoirs intact. As an alternative approach, antibodies targeting virion envelope glycoproteins have been developed to neutralize cell-free virus entry (5, 6, 7). However, dissemination within a host likely depends primarily on direct cell-to-cell spread (8, 9, 10, 11, 12), which is resistant to neutralizing antibodies (13). These limitations underscore the need for novel therapeutic strategies that can address both the lytic and latent phases of HCMV infection.

The recent studies have demonstrated that HCMV encodes effective countermeasures against a spectrum of immune responses. This arsenal of immunomodulatory functions is likely a reflection of the natural history of the virus, providing the capacity to establish lifelong infections of the host as well as to reinfect people with an existing infection despite the presence of a substantial immune response. The complexity of these immunological interactions is being studied extensively. We and others have showed that HCMV has developed many sophisticated mechanisms targeting host immunity, thus the HCMV has become a paradigm for viral immune evasion. Suffice it to say that HCMV-encoded gene functions target antigen presentation by major histocompatibility complex class I and class II molecules, utilize cytokine mimicry to exert paracrine functions against immune cells, and encode proteins that antagonize the range of innate immune responses directed against the virus. We have been studying several of viral immune modulatory genes over the last decade, primarily focusing on those that target and/or intersect with Ig-like and TNF family signaling. More insightfully, the natural killer (NK) cells are crucial for virus control (14) resulting in HCMV having an impressive arsenal of encoded immune evasins that act to suppress NK cell activation through the manipulation of ligands for activating and inhibitory receptors (15, 16, 17, 18, 19, 20). Importantly, recent studies have revealed how disrupting the function of a single HCMV immune-modulating gene UL141 can tip the balance in favor of host defense. UL141 restricts the surface expression of CD155 and CD112, which activate NK *via* DNAM-1 and CD94 (21). UL141 also binds and inhibits expression of the TRAIL death receptors to block NK-mediated killing by this TNF family cytokine (19, 22). UL141 together with HCMV US2 were found to be the effective modulators of multiple immune-related pathways and act as a multifunctional degradation hub that inhibits the migration, immune recognition, and killing of HCMV-infected cells (23). In addition, HCMV UL141 was reported to affect viral DNA replication (24) while interacting with CELF5, highlighting the polyfunctionality that a single HCMV immunomodulatory protein can have. To gain a more comprehensive understanding of UL141 antigen potential, Vlahava *et al.* (25) isolated human monoclonal antibodies specific for UL141 from a seropositive donor. Cloned antibodies targeting a single antigen (UL141) were sufficient to mediate antibody-dependent cellular cytotoxicity against HCMV infected cells, even at low concentrations.

The above evidence suggests that UL141 could serve as promising target for novel therapeutics development, thus we sought to develop a small UL141 antagonist as the direct-acting drug. In general, this class of antiviral drugs usually target one of the essential components of the virus itself, while, on the other site, the host-directed drugs target a host process required for viral replication (26). Leveraging our crystal structure of HCMV UL141 (17, 18) with detailed receptor binding interface, we further aimed to map its potential for small molecule interactions. Iminosugars seem to be promising candidates for this antivirals development (27) as their antiviral activity as host-directed drugs have been proved against a range of enveloped viruses (28, 29, 30, 31, 32, 33, 34, 35, 36, 37).

Therefore, the mounting evidence of UL141's direct involvement in HCMV entry (1), coupled with the above characteristics, suggests that UL141 possesses several critical functions that enable HCMV dominance (17, 19, 21, 23, 38, 39). Given that UL141 is emerging as a versatile viral protein, it represents a promising candidate for the development of novel therapeutic strategies. In this study, considering the known relationship between glycosidase inhibition and antiviral activity, selected glycomimetics were screened against this viral protein to develop the UL141 antagonist as the direct-acting drug. Within the study, the structure of the hit compound was further modified with the aim to improve the antagonist potency and understand the binding mechanism between the iminosugar and the viral protein. Based on our recent crystal structures (17, 18) and molecular modeling, the binding site of the *de novo* synthetic antagonist on the surface of the UL141 protein was proposed and additionally, verified by mutational analysis.

## Results

### Synthesis of glycomimetics

**Figure 1:**
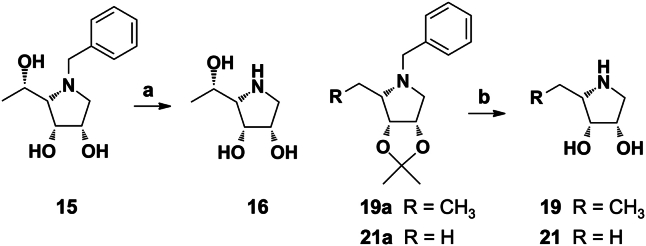
**The synthesis of pyrrolidine hydrochlorides 16, 19, and 21.** Reagents and conditions: a, 1. H_2_, Pd/C, MeOH, rt, 2 h, then conc. HCl, 94%; (b) 1. H_2_, Pd/C, MeOH, rt, 2 h, 2. conc. HCl, 0 to 40 °C, 2 h, 79% for 19, 66% for 21.

**Figure 2:**
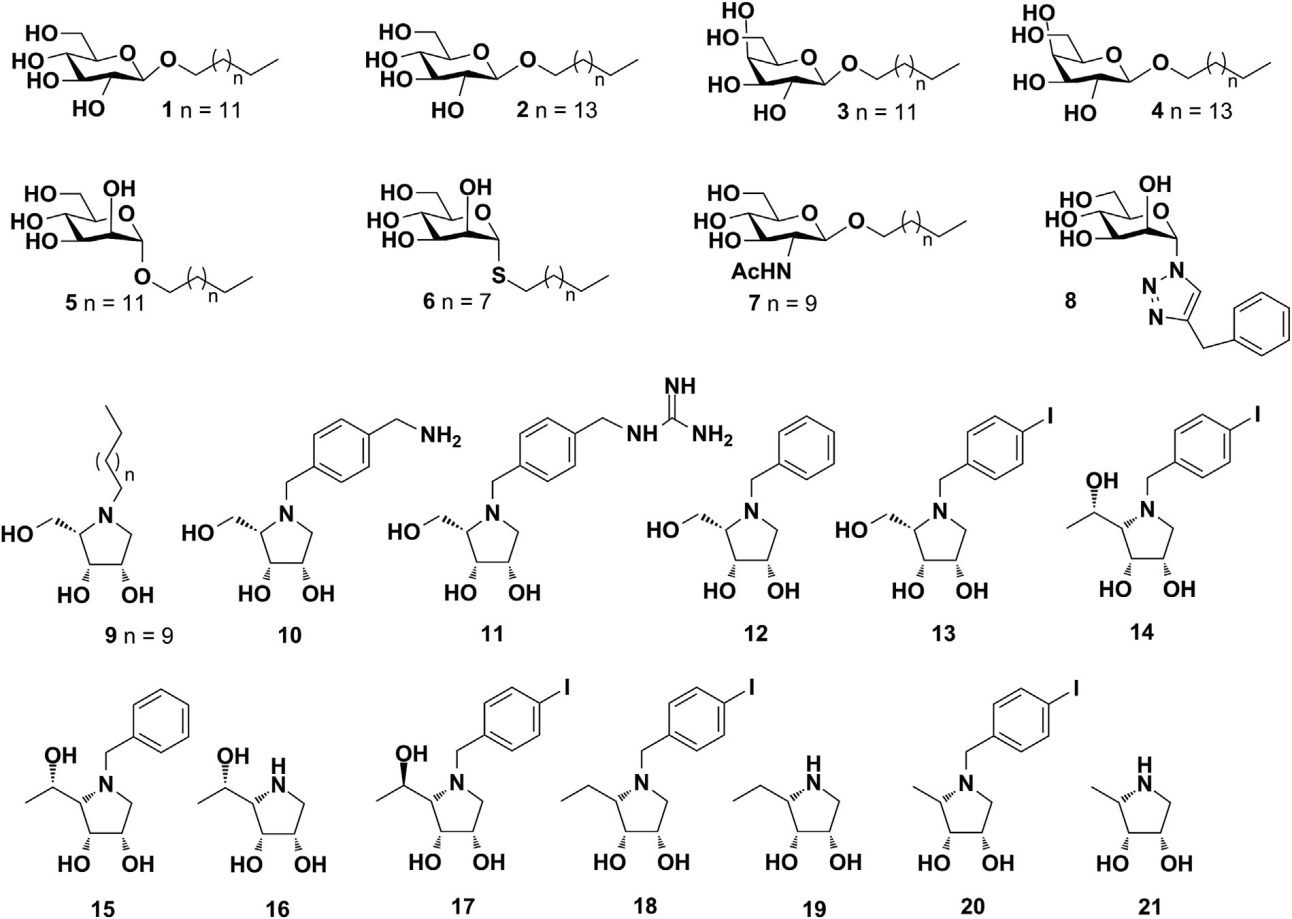
**Chemical structures of compounds 1 to 21 used in bioassays targeting UL141.** The variety of compounds, including glycosides 1 to 7; glycosyltriazole 8; iminosugars 9 to 21 were screened for the ability to block the UL141–TRAIL-R2 complex formation.

Synthesis and characterization of glycomimetics including a brief reaction scheme are described and illustrated in Experimental procedures and Supplementary methods. The synthesis of glycosides 1 to 5 (40), 6 (41), 7 (42), glycosyltriazole 8 (43), and iminosugars 9 to 15, 17, 18, and 20 (44, 45, 46, 47) were already reported in our previous studies. The pyrrolidines 16, 19, and 21 in hydrochloride form were synthesized from the corresponding benzylated intermediates (Fig. 1). Namely, the hydrochloride of the compound 16 was obtained in high yield from the *N*-benzyl derivative 15 after hydrogenolysis and treatment with concentrated HCl. Similarly, hydrogenolysis of 19a (45) and 21a (45) and subsequent removal of acetonide protective group under acidic condition gave hydrochlorides 19 and 21. Complete set of 21 derivatives was employed in this study for screening toward viral protein (HCMV UL141) in the presence of human receptor (TRAIL-R2). The structures of compounds 1 to 21 are depicted in Figure 2.

### UL141 (full length) tightly binds to recombinant human TRAIL-R2

UL141 is a protein encoded by the HCMV genome. It is classified as a type I transmembrane glycoprotein, containing a short cytoplasmic domain at its C-terminus. Recent structural analyses have revealed that UL141 features a V-type immunoglobulin superfamily fold in its N-terminal domain (17, 18), suggesting its interaction with host cell proteins and subsequent influence on various cellular processes (including viral entry). Our previous studies demonstrated a direct interaction between UL141 and both TRAIL-R2 and TRAIL-R1, as well as CD155, highlighting the structural versatility of the UL141 immunoglobulin fold in receptor binding (17, 18, 19). This discovery underscored the unexpected pleiotropic role of UL141 in modulating receptor binding and host immunity.

**Figure 3:**
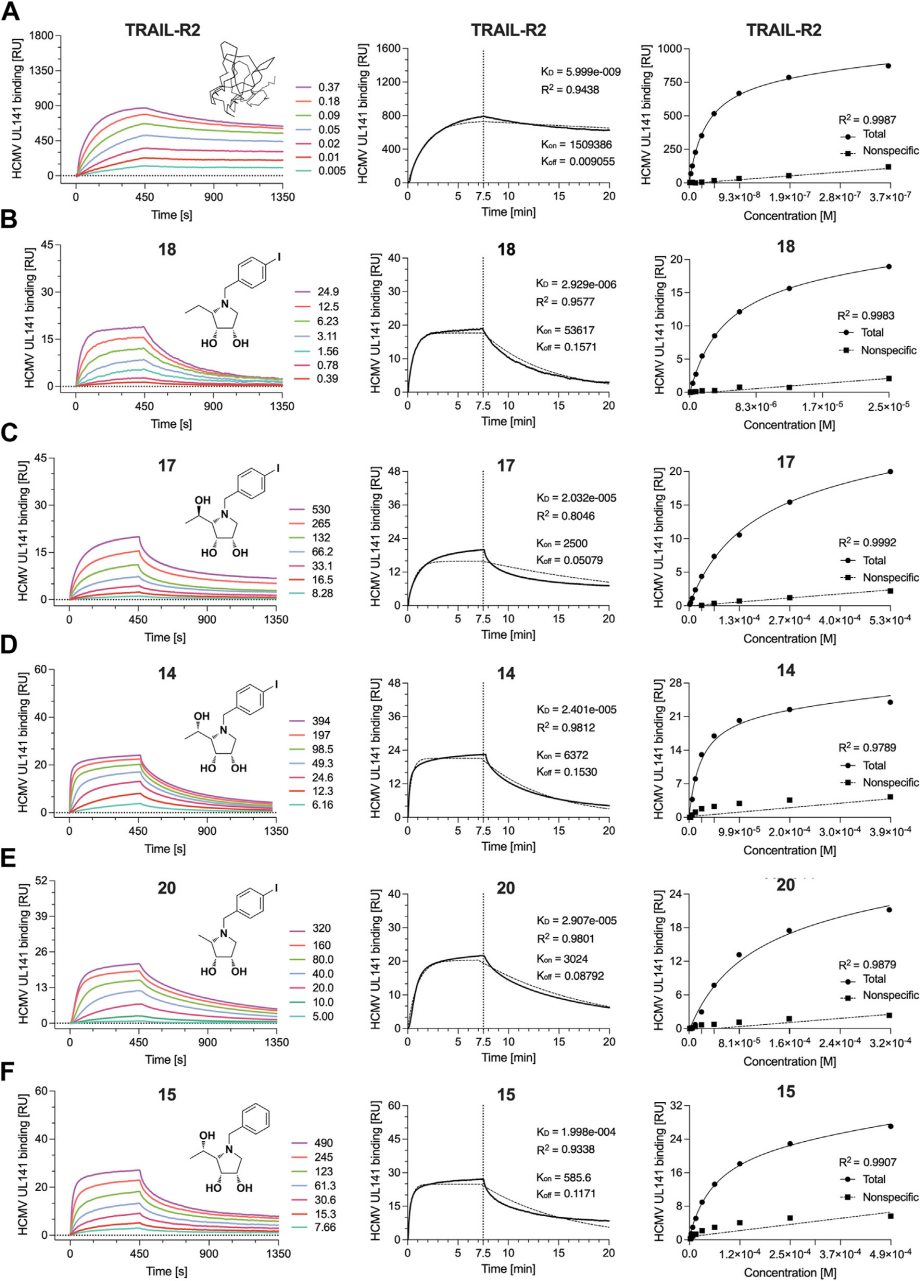
**Surface plasmon resonance analysis was conducted to evaluate the binding interactions between HCMV UL141 and TRAIL-R2, as well as compounds 14, 15, 17, 18, and 20.** The *left panels* display colored sensorgrams for each kinetic experiment (*A*–*F*), with indicated analyte concentrations and their chemical structure or simplified molecular structure in case of TRAIL-R2 (*A*) and for individual compounds (*B*–*F*). Kinetic parameters were determined from 2 to 3 independent experiments by fitting the experimental data (*bold lines*) using a 1:1 Langmuir binding model. The fit is shown by *dotted lines* for the highest measured concentration in the *middle panels*. The resulting binding constant (K_D_, M), association rate (k_on_, M^−1^s^−1^), and dissociation rate (k_off_, s^−1^) are shown along with the corresponding R-squared (R^2^) values, indicating the quality of each fit (*A*–*F*). The *right panels* illustrate the distinction between specific and nonspecific binding of the same experiment.

In prior experiments, interactions were studied using a recombinantly expressed UL141 ectodomain (amino acids 30–217 and 30–279), both lacking short C-terminal helical domain. These constructs, expressed and isolated from mammalian or insect cells, included C-terminal tags. To assess whether the full-length UL141 (incl. short helical domain) exhibits similar binding properties, we expressed and purified the full-length recombinant UL141 (amino acids 30–300), both with and without an N-terminal octa-histidine tag (UL141-FL-8H and UL141-FL, respectively), as well as a C-terminal fusion protein with the Fc region of human IgG_1_ (UL141-FL-Fc). Similarly, TRAIL-R2 was recombinantly prepared as a fusion protein (TRAIL-R2-Fc) according to Nemčovičová *et al.*, 2013 (17). The affinity tags were cleaved as required, depending on the specific applications. The previous UL141 ectodomain expression exhibited poor protein stability, complicating expression and crystallization efforts (17, 18). However, modifications to the length of the expression construct (addition of a C-terminal helical domain) and the repositioning of tags (from C- to N-terminal) led to improved protein quality and stability in solution, enabling more robust downstream applications. The surface plasmon resonance (SPR) binding assay demonstrated a tight interaction between full-length UL141 and TRAIL-R2 (Table 1A and Fig. 3*A*), with an affinity of 5.99 nM, consistent with our previous findings on the UL141 ectodomain (K_D_ = 5.96 × 10^−9^ M). Notably, other kinetic parameters closely aligned with those observed in earlier experiments, showing a relatively mild association rate (k_on_) but slow dissociation (k_off_). Small differences in k_on_ and k_off_ values can be attributed to the different SPR immobilization techniques used: in the ectodomain study, UL141 was immobilized *via* its large Fc-fusion tag, while in the current experiment, biotinylated UL141 (without tags) was immobilized *via* streptavidin on the chip. The presence of the larger tag in the earlier study may have influenced the overall binding kinetics. The interaction between full-length UL141 and TRAIL-R2 was confirmed to be very tight (6 nM), serving as a positive control for subsequent SPR-binding experiments presented in this report.

**Table 1:** Binding kinetics measured by surface plasmon resonance (SPR) calculated from data presented in Figure 2

	Immobilized (ligand)	In solution (analyte)	R_max_	R^2^ (highest conc.)^b^	K_D_ [M]	k_on_ [M^−1^s^−1^]	k_off_ [s^−1^]	Ref.
A^a^	UL141	TRAIL-R2	872	0.9438	(5.999 ± 0.111) × 10^−9^	(1.51 ± 0.028) × 10^6^	(9.05 ± 0.006) × 10^−3^	
	TRAIL-R2-Fc	UL141	41	n.d.	5.960 × 10^−9^	1.21 × 10^4^	7.21 × 10^−5^	(19)
	UL141-Fc	TRAIL-R2	39	n.d.	21.40 × 10^−9^	2.64 × 10^5^	5.64 × 10^−3^	(17)
B	UL141	cmp 18	19	0.9577	(2.929 ± 0.073) × 10^−6^	(5.36 ± 0.003) × 10^4^	(1.57 ± 0.039) × 10^−1^	
C	UL141	cmp 17	20	0.8046	(2.032 ± 0.065) × 10^−5^	(0.25 ± 0.008) × 10^4^	(0.51 ± 0.001) × 10^−1^	
D	UL141	cmp 14	24	0.9812	(2.401 ± 0.066) × 10^−5^	(0.63 ± 0.002) × 10^4^	(1.53 ± 0.041) × 10^−1^	
E	UL141	cmp 20	21	0.9801	(2.907 ± 0.084) × 10^−5^	(0.30 ± 0.007) × 10^4^	(0.88 ± 0.015) × 10^−1^	
F	UL141	cmp 15	27	0.9338	(1.889 ± 0.069) × 10^−4^	(0.06 ± 0.002) × 10^4^	(1.17 ± 0.004) × 10^−1^	

a Values from reference (17, 19).

b R^2^ indicates how data of the highest analyte concentration fit to the 1:1 Langmuir binding model.

### UL141 antagonists may potentially prevent TRAIL-R2 binding

**Figure 4:**
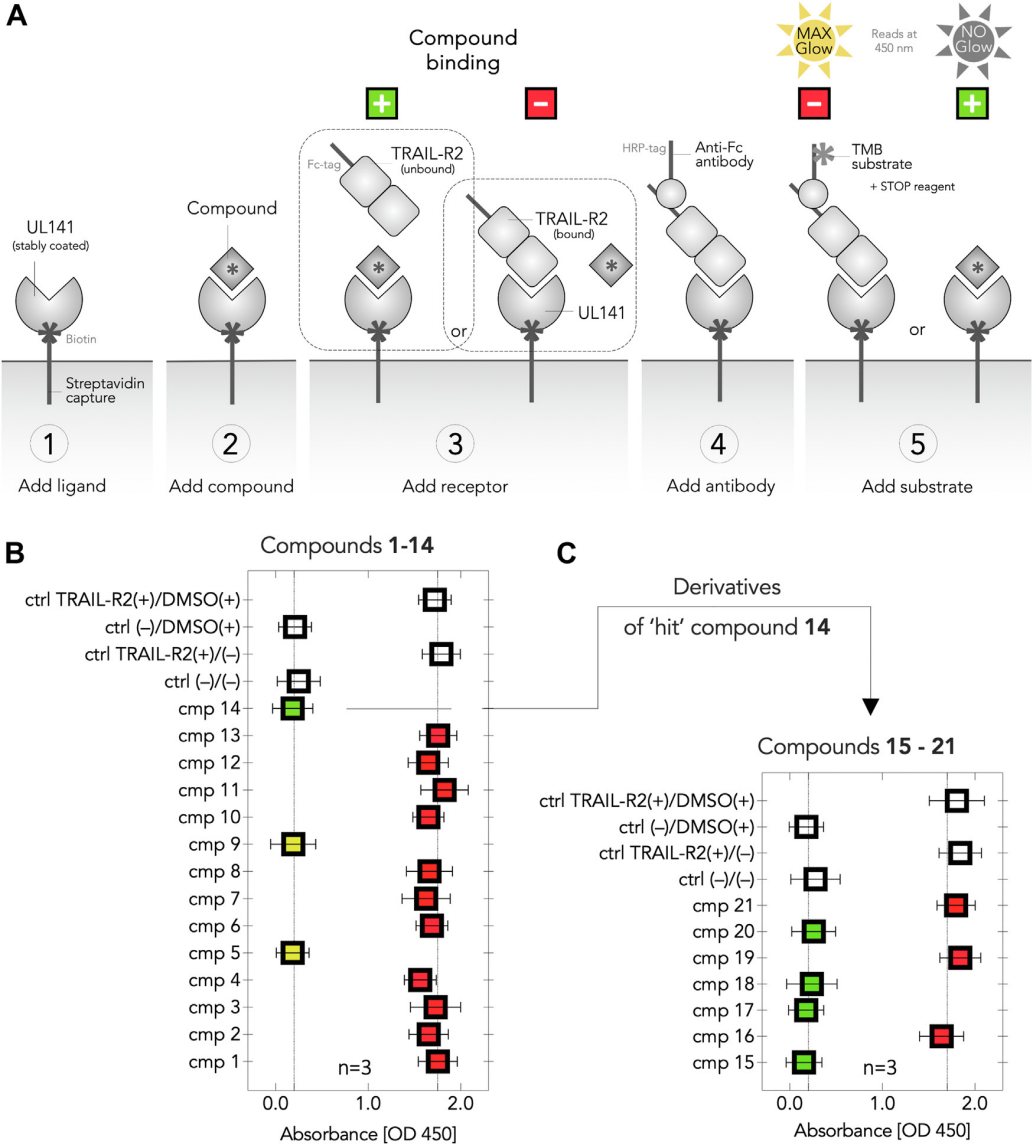
**ELISA-based screening of compounds (cmp) 1 to 21 for their ability to block UL141–TRAIL-R2 complex formation *in vitro*.** The principle of the binding assay is demonstrated in the *upper* scheme (*A*). Initial screening was performed with first fourteen cmp 1 to 14 (*B*), and seven additional cmp 15 to 21 were screened later using the same assay (*C*). *Green* and *yellow* colors indicate a positive antagonist of UL141 with the ability to block TRAIL-R2 binding, while *yellow* also indicates unfavorable compound toxicity. *Red* color indicates binding to TRAIL-R2, meaning no disruptive effect on complex formation by the antagonist. Transparent controls (ctrl) represent DMSO (+/−) and/or TRAIL-R2 (+/−) in the absence of any compound. The number of biological replicates is indicated.

Initially, we evaluated the ability of the fourteen compounds (1–14) to inhibit the formation of the UL141–TRAIL-R2 complex using *in vitro* assays, including ELISA and SPR. To evaluate potential UL141 antagonists, we developed a streamlined ELISA-based binding assay. This assay utilized an HRP-labeled antibody to detect the bound TRAIL-R2 receptor, with TMB substrate serving as the final detection reagent (Fig. 4). In this assay, biotinylated UL141 is immobilized onto microplate wells through streptavidin-coated dynabeads. TRAIL-R2 in the sample binds to the immobilized UL141, and the amount of bound TRAIL-R2, which reflects its binding activity, is detected using an anti-Fc (HRP-labeled) antibody. This is followed by a colorimetric reaction, measured through a microplate spectrophotometer at 450 nm, corresponding to the absorbance of the stopped TMB substrate reaction. The binding activity of TRAIL-R2 is proportional to the optical density measured at this wavelength. To assess potential antagonists, the UL141-coated wells were pre-incubated with the test compounds (step 2) (Fig. 4*A*) prior to the addition of TRAIL-R2 (step 3). The results showed that treatment with three out of the fourteen compounds (5, 9, and 14) prevented TMB signal generation, indicating disruption of TRAIL-R2 receptor binding (Fig. 4*B*, green and yellow squares). In contrast, the remaining compounds (1–4, 6–8, and 10–13) did not interfere with the formation of the TRAIL-R2–UL141 complex, as evidenced by TMB signal detection (Fig. 4*B*, red squares). These findings suggest that compounds 5, 9, and 14 may inhibit the formation of the TRAIL-R2–UL141 complex, implying that direct interaction between these compounds and UL141 could play a role, and this was further explored in next experiments.

### Molecular optimization of the hit structure

**Figure 5:**
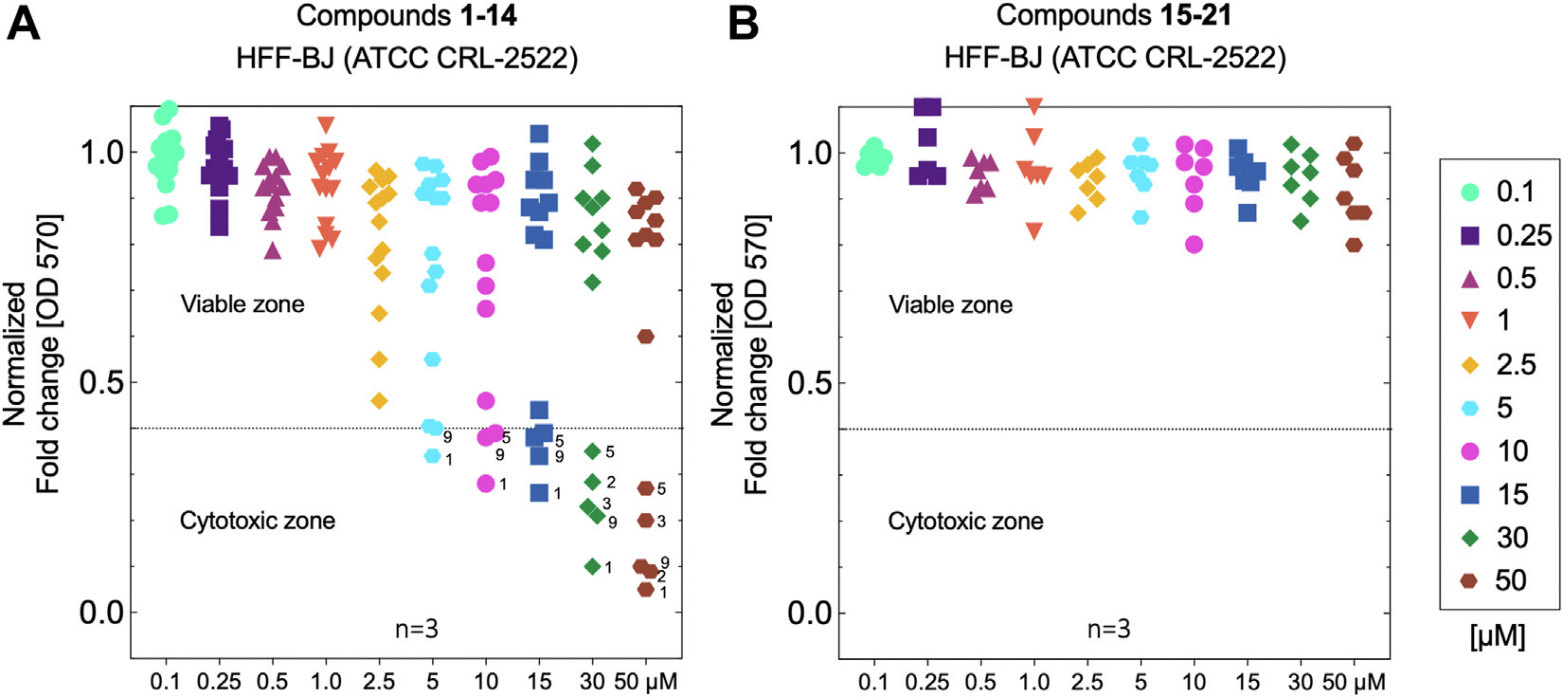
**Apoptotic responses measured spectrophotometrically by OD at 570 nm (formazan absorption) induced by the compounds studied in human foreskin fibroblasts HFF-BJ (ATCC CRL-2522 cells).** Cells were treated with the indicated concentrations of compounds 1 to 14 (*A*) and compounds 15 to 21 (*B*) for 72 h. Cell viability as visualized by fold change of formazan-positive cells normalized to the untreated control and DMSO. Viability reduced below 40% represents the cytotoxic zone, below the dotted line in each plot. The number of biological replicates is indicated.

**Figure 6:**
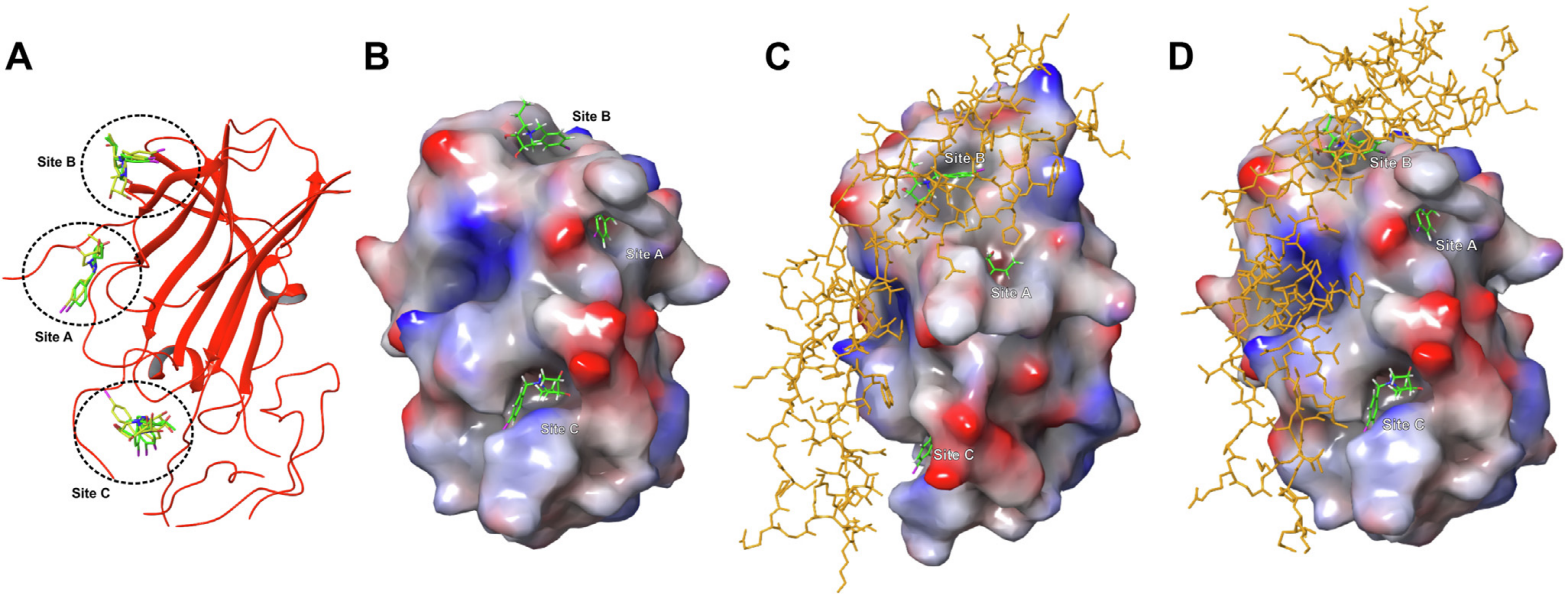
**Molecular modeling of active UL141 antagonists.** Docked 14 (*yellow*) and 18 (*green*) in UL141 glycoprotein (*red* ribbons). Preferred binding sites are marked with circles (site A, B, and C) (*A*). Docked 18 (*green*) in UL141 (VDW surfaces) visualized with electrostatic potential (*red* surfaces are with more negative values and *blue* ones are with more positive values) (*B*). Docked 18 (*green*) in UL141 glycoprotein (VDW surfaces). Preferred binding sites of 18 are marked as site A, B, and C. Human TRAIL-R2 protein (*orange*, tubes) is superimposed as it was bound in an X-ray complex with UL141 (PDB ID: 4I9X) (*C*). A side view of the same complex (*D*).

**Figure 7:**
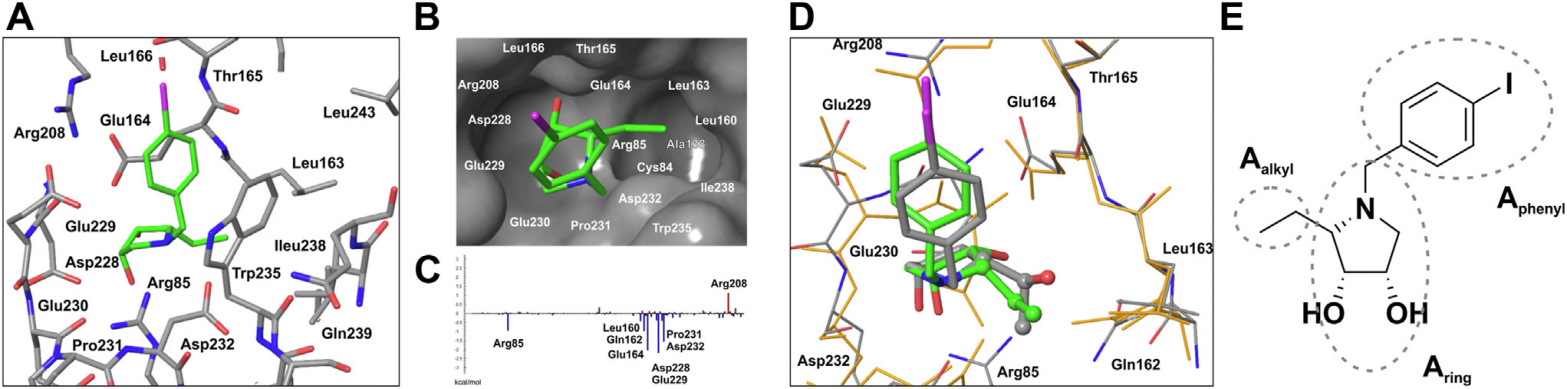
**FMO interaction energy analysis of site C.** The antagonist 18 bound at site C (pose 1), hydrogens are not visualized for clarity (*A*). Details of a hydrophobic pocket consisted of Cys84, Ala138, Leu160, Leu163, and Ile238 (*B*). FMO pair interaction energies (Δ*E*_Aalkyl:UL_^int^) (in kcal mol^−1^) between the 2-ethyl fragment of the antagonist 18 and sites C of UL141 protein. The most significant Δ*E*_A:UL_^int^ are marked (*C*). Superposition of the QM/MM optimized complex [14(pose 1):UL141(site C)] (14 in *gray* and UL141 in *orange*) with [18(pose 1):UL141(site C)] (18 in *green* and UL141 with carbon in *gray*, oxygen in *red*, and nitrogen in *blue*). The 2-ethyl moiety of 18 and 2-[(*S*)-1-hydroxyethyl] moiety of 14 are visualized in ball-and-stick atom representation. The presence of the bulkier 2-[(*S*)-1-hydroxyethyl] moiety shifted the binding position of the pyrrolidine ring of 14 in site C of UL141 compared to 18 (*D*). Schematic decomposition of the structure of the antagonist 18 for the FMO-PIEDA calculations (*E*).

Based on the results of the initial ELISA screening, compound 14 was identified as the primary “hit” structure, alongside two other compounds 5 and 9. However, these two compounds were excluded from further analysis due to an unfavorable level of cytotoxicity observed in cellular assays (Fig. 5*A*), as detailed in the following paragraph. Consequently, compound 14, with systematic name of (2S,3R,4S)-2-((S)-1-hydroxyethyl)-1-(4-iodobenzyl)pyrrolidine-3,4-diol, was selected for subsequent structural optimization aimed at developing enhanced UL141 antagonists (Fig. 6*A*). Based on its chemical structure, the most significant was to investigate the effects of 1-aryl and 2-alkyl substituents (Fig. 7*E*) on binding to the UL141 and to improve its pharmacological properties. Therefore, several derivatives were designed, including those with 1-benzyl, 1-(4-iodobenzyl), 2-methyl, 2-ethyl, 2-[(*R*)-1-hydroxyethyl], and 2-[(*S*)-1-hydroxyethyl] substituents. These optimized derivatives (designated as structures 15–21) were *de novo* synthesized as described above or obtained from our previous studies (Fig. 2). The compounds were screened using the same ELISA-binding assay to evaluate their ability to prevent the TRAIL-R2–UL141 complex formation, and their cellular cytotoxicity was also assessed (Fig. 5*B*). The ELISA results clearly demonstrated that treatment with compounds 15, 17, 18, and 20 prevented TMB signal generation (Fig. 4*C*, green squares), suggesting disruption of TRAIL-R2 receptor binding. In contrast, compounds 21, 19, and 16 did not interfere with the formation of the TRAIL-R2–UL141 complex, as indicated by the presence of a TMB signal (Fig. 4*C*, red squares). These findings suggest that compounds 15, 17, 18, and 20 may inhibit complex formation similarly to compound 14, as observed in previous assays. Collectively, these results imply that direct binding to UL141 could be a contributing factor, therefore this was tested in subsequent SPR experiments.

### Cellular toxicity assessment of new compounds

To ensure that the selected molecules (potential UL141 antagonists) do not adversely affect cells or tissues, a cellular cytotoxicity assessment was conducted at the beginning. Human foreskin fibroblasts (HFF), a type of primary fibroblast derived from neonatal foreskin tissue, were chosen for this bioassay due to their distinct advantages in studies involving HCMV. For instance, the HHF cells provide a relevant and effective platform due to their unique susceptibility to HCMV infection, ability to mimic human immune responses, and relevance to clinical scenarios. To determine the actual cytotoxicity *in vitro* of all studied compounds 1 to 21 (Fig. 2), we tested cellular viability using MTT assay. HFF cells were incubated with each compound at concentrations ranging from 0.1 to 50 μM and any possible cytotoxic effect was monitored for 72 h. Tested compounds were dissolved in dimethyl sulfoxide (DMSO). None of the tested compounds showed any signs of insolubility. The cytotoxic effect (CTE) is represented here as the concentration that causes a reduction of more than 40% in the number of viable cells. Interestingly, most of the experimental compounds were relatively well tolerated in selected cell line, except some cases (Fig. 5*A*). The most pronounced cytotoxicity was observed with compounds 1, 5, and 9, which exhibited CTE even at lower concentrations in tested HFF cell line (starting at approximately 2.5–5 μM). Compounds 2 and 3 were deemed cytotoxic at considerably higher concentrations, around 10 to 15 μM. Moreover, our findings for compounds 1 to 4 align with previously reported antiproliferative properties in various cancer cell models (*e.g.*, CCRM-CEM, CEM-DNR) (40, 46). In addition, compounds 1, 3, 5, and 9 demonstrated cytotoxicity, with apoptotic properties observed at concentrations beginning at 5 μg/ml (approx. 10–13 μM) when tested on A549 and VERO-E6 cells (data not shown). However, due to the CTE observed in HFF cells, compounds 5 and 9, despite their demonstrated antagonist activity against UL141, were excluded from further evaluation.

In conclusion, only compounds that exhibited cytotoxicity at higher concentrations (CTE >15 μM) or showed no cytotoxic effects within the tested concentration range (Fig. 5*B*) were advanced for further antagonist efficacy testing and binding constant estimation toward UL141. Conversely, the selectivity index (SI = CTE/K_D_) for the most active compounds (14–15, 17–18, 20) remained moderate, as no cytotoxicity was detected even at the highest tested concentrations. This may indicate that the compound is selective and has a wide safety margin.

### UL141 binds five potential glycomimetics with micromolar affinities

The streamlined ELISA results indicated that glycomimetics binding to UL141 may be an interfering factor when no interaction to TRAIL-R2 was detected. Therefore, we sought to determine whether these small glycomimetics could bind directly to UL141 and whether their binding sites might overlap with or interfere with the TRAIL-R2–binding sites. The binding kinetics of UL141 to either TRAIL-R2 (used as a positive control) or compounds 14, 15, 17, 18, and 20 were subsequently measured using SPR. The biotinylated ligand UL141 was successfully immobilized on sensor chip *via* streptavidin and the increasing concentrations of analytes (2-fold dilutions) were tested. The binding kinetics of UL141 with glycomimetics 14, 15, 17, 18, and 20 demonstrated comparable characteristics (Fig. 3 and Table 1), particularly in their association rates, which reflect the speed of complex formation. The relatively low K_on_ values, ranging from 0.06 to 5.36 × 10^4^ M^−1^s^−1^, suggest slow to very slow association. In contrast, the higher K_off_ values, ranging from 0.51 to 1.57 × 10^−1^ s^−1^, indicate rapid dissociation of the glycomimetics from the complex with UL141. Notably, k_off_ > 10^−2^ is typically associated with fast dissociation events. Interestingly, the binding kinetics of UL141 to either TRAIL-R2 or glycomimetic 18 differed significantly (A vs. B, in Table 1 and Fig. 3). UL141 bound to compound 18 with slower association rate, while dissociation was 17-times faster, resulting in a nearly 500-fold higher equilibrium binding affinity (K_D_) compared to TRAIL-R2. As expected, these differences underscore the significantly stronger binding affinity of UL141 to TRAIL-R2 compared to small molecule like 18, which exhibits considerably weaker binding and more rapid dissociation. The observed kinetic differences suggest that UL141 may engage distinct binding sites to bind TRAIL-R2 compared to structurally smaller molecule like 18 or suggested different binding mechanisms (*e.g.*, hydrogen bonding and/or electrostatic interactions combined with induced fit or multivalent binding). To test these hypotheses, we conducted *in silico* molecular modeling studies focusing on the two most active antagonists, 14 and 18. Subsequently, mutational analysis was performed on suggested surface residues of UL141. The effects of these mutations on the binding affinity of compound 18 were evaluated to further validate the proposed interactions.

### Molecular modeling of the two most active antagonists

Based on the findings from the SPR and ELISA experiment, which demonstrated that six glycomimetics can directly bind to UL141 and thereby inhibit complex formation, we aimed to assess whether their binding sites might overlap with or interfere with the TRAIL-R2 binding site, as revealed by the crystal structure (PDB ID: 4I9X). These findings prompted a deeper investigation into the binding mechanisms, leading us to explore the possibility of distinct binding sites for UL141 when interacting with glycomimetics. Therefore, the hit structure 14 and the most active compound 18 from optimized studies were used for a comprehensive molecular docking. Compounds 14 and 18 were docked into UL141 virial protein, then hybrid QM/MM (quantum mechanics/molecular mechanics) geometry optimizations of the antagonist–protein complexes and fragmented molecular orbital–pair interaction energy decomposition analysis (FMO-PIEDA) calculations were performed. The main aim was to describe a structure and an interaction pattern between the antagonist and the protein. Structure of 18 differs from 14 at the C2 position, where 2-[(1*S*)-1-hydroxyethyl] group in 14 was substituted by 2-ethyl group in 18. We wanted to understand why the omission of one hydroxyl group in 18, compared to 14, increased the ability of 18 to block UL141 (*K*_D_ decreased from 24 μM to 2.9 μM for 14 and 18, respectively). The compounds 14 and 18 were docked on a surface of UL141 which is a part of the binding interface between UL141 and TRAIL-R2 (17) as it was described in an X-ray structure of the protein complex (PDB ID: 4I9X). This surface was divided into three docking grids, the first one centered around Arg82 amino acid residue, the second one centered around Tyr148, and third one centered around Trp235. The preferred binding sites of the antagonists 14 and 18 are depicted in Figure 6 and were named as site A, B, and C. In the site A, the antagonist binds closely to Asp91 (not to Arg82 as was expected), in site B to Tyr148 and Arg 146, and in site C to Trp235 and Asp232. Docking scores of 14 and 18 are in the range of 4.3 to 5.9 kcal mol^−1^ [docking with standard precision] and 4.2 to 6.8 kcal mol^−1^ [docking with extra precision] predicting site C as slightly preferable for 18 and both sites B and C as preferable for 14 (Table 2). For subsequent QM calculations, only the best docked positions of the antagonists (pose 1 by docking score, ΔG^dock^) were used; however, the second best positions (pose 2) were also included for sites B and C, as the difference in docked positions 1 and 2 of the antagonist at these binding sites was significant.

**Table 2:** Docking scores and fragment interaction energies of the UL141 antagonists (14 and 18)

Position in UL141	conf	pK_a_	ΔG^dock^(SP)	ΔG^dock^(XP)	ΔE_A:UL_^int^	ΔE_Aring:UL_^int^	ΔE_Aphenyl:UL_^int^	ΔE_Aalkyl:UL_^int^
Structure 14								
Site A (pose 1)	^4^E	10.1	−5.3	−4.7	−164.2	−112.5	−28.6	−23.1
Site B (pose 1)	E_5_/^4^E	11.4	−5.1	−6.8	−115.6	−80.7	−11.6	−23.3
Site B (pose 2)	^1^E	10.4	−4.4	−5.0	−92.5	−76.8	−10.8	−4.9
Site C (pose 1)	E_5_	10.5	−5.9	−4.9	−203.0	−163.0	−14.7	−25.3
Site C (pose 2)	^4^E	10.3	−4.8	−5.0	−169.5	−129.0	−19.1	−21.4
Structure 18								
Site A (pose 1)	E_5_	9.9	−4.5	−4.6	−181.8	−152.5	−22.3	−7.0
Site B (pose 1)	^1^E/E_2_	10.4	−4.4	−4.3	−95.1	−84.8	−11.4	1.1
Site B (pose 2)	^1^E	10.5	−4.3	−4.2	−82.9	−72.2	−11.1	0.4
Site C (pose 1)	^4^E	8.7	−4.6	−6.4	−208.9	−182.3	−18.4	−8.2
Site C (pose 2)	E_5_	8.1	−4.5	−5.8	−211.8	−178.3	−29.0	−4.6

Docking score (ΔG^dock^) calculated with Glide program [standard (SP) and extra precision (XP)] (in kcal mol^−1^); total interaction energy (ΔE_A:UL_^int^) between the antagonist 14 (and 18) and the UL141 protein (sites A, B and C) calculated with the FMO-PIEDA method at the MP2//M06-2X level. The interaction energies (ΔE_A:UL_^int^ = ΔE_Aring:UL_^int^ + ΔE_Aphenyl:UL_^int^ + ΔE_Aalkyl:UL_^int^) between three fragments of the antagonist (A_ring_, A_phenyl_, and A_alkyl_, Fig. 7*E*) and UL141 protein as well as the pyrrolidine ring conformations of the bound antagonists and calculated pK_a_ values of amino group of the antagonists are also compiled.

Additional FMO pair interaction energy analysis at the QM level (Fig. 7, *A*–*C*) predicted for site C, the most favorable total interaction energy between UL141 protein and antagonist 18 [ΔE_A:UL_^int^ = −208.9–(−211.8) kcal mol^−1^, Table 2]. Predicted ΔE_A:UL_^int^ for site A and B was with a smaller negative value [Δ*E*_A:UL_^int^ = −82.9–(−181.8) kcal mol^−1^ for 18] (Table 2 and Fig. S1). The similar energy trends were also found for the antagonist 14 [ΔE_A:UL_^int^ = −169.5 –(−203.0) kcal mol^−1^ (site C), −164.2 kcal mol^−1^ (site A), and −92.5–(−115.6) kcal mol^−1^ (site B)]. Different docking positions (pose 1 and 2) of the antagonists for each binding site did not change energetic preferences of the antagonist for binding sites A, B and C, that is, for the site C, ΔE_A:UL_^int^ is still the most favorable even when both antagonist positions 1 and 2 are included in FMO-PIEDA calculations. These calculations suggest that site C could be the most likely binding site on the surface of UL141 for glycomimetic compounds studied in this work. This was further investigated in subsequent experiments using mutational and binding analyses by SPR, where the interacting amino acids—Asp91, Glu92, Arg82, Arg80, and Asp37 (site A); Tyr148 and Arg146 (site B); and Arg85, Leu163, Glu164, Pro231, Trp235, and Asp232 (site C)—were later mutated to alanine.

**Figure 8:**
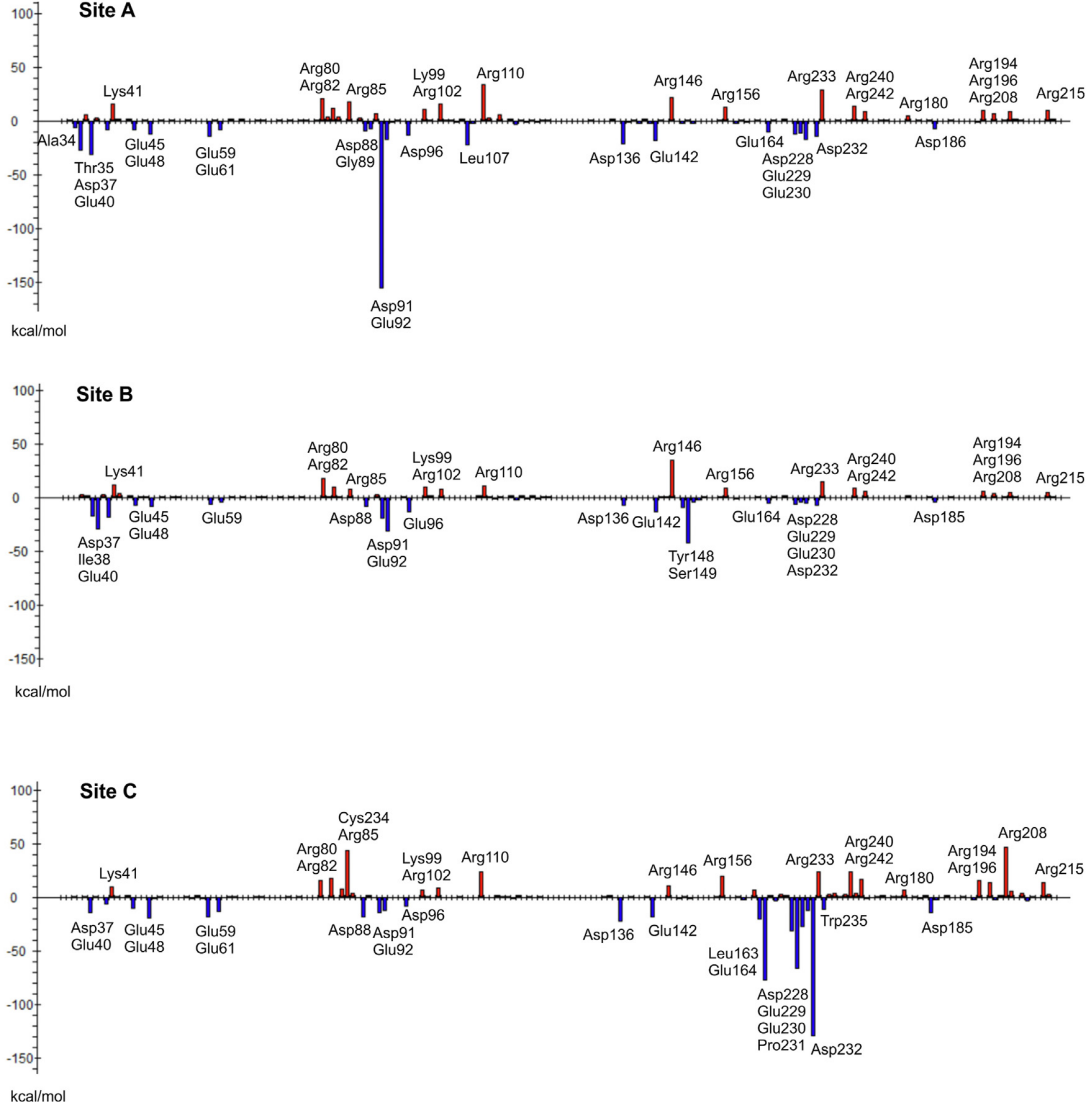
**FMO pair interaction energies (Δ*E*_A:UL_^int^) (in kcal mol^−1^) between the antagonist 18 and UL141 protein (binding sites A, B, and C, poses 1).** The most significant Δ*E*_A:UL_^int^ are marked.

Detailed interaction energy patterns of 18 and 14 are compiled in Figures 8 and S2 of ESI, respectively. As can be seen, the major contributors to interactions are negatively charged amino acid residues of UL141 as aspartic or glutamic acids; in opposite, the major contributors to repulsions are positive charged residues as arginine. The structure of both antagonists consists of the pyrrolidine ring, which prefers protonation form at the binding sites on the surface of UL141. According to p*K*_a_ calculations, p*K*_a_ values for amino group of the pyrrolidine ring of the bound antagonist are in the range of 8.1 to 10.5 (for 18) and 10.1 to 10.5 (for 14) (Table 2). Thus, the protonation form of the bound antagonists on the surface of UL141 favorized the interactions with negatively charged amino acid residues of UL141 and in opposite, increased repulsions with positively charged amino acid residues. Because site C was predicted as the most probable binding site of the antagonists on the surface of UL141, only the interaction pattern [antagonist 18: UL141(site C)] will be further described in detail (Figs. 7, *A*–*C* and 8). The structure of 18 was divided to three structural moieties: the pyrrolidine ring (A_ring_) with three polar groups (two hydroxyl and one amino group), the aromatic 1-(4-iodophenyl) moiety (A_phenyl_), and hydrophobic 2-ethyl chain (A_alkyl_) (Fig. 7*E*). The interaction energies between these structural moieties of the antagonist and amino acid residues of UL141 were evaluated as Δ*E*_Aring:UL_^int^, Δ*E*_Aphenyl:UL_^int^, and Δ*E*_Aalkyl:UL_^int^ (Table 2). On the side of the antagonist, the major contributor to the interaction energy (Δ*E*_A:UL_^int^) is the pyrrolidine fragment (Δ*E*_Aring:UL_^int^ presents 84–87% of Δ*E*_A:UL_^int^). Δ*E*_Aphenyl:UL_^int^ and Δ*E*_Aalkyl:UL_^int^ presents only 9 to 14% and 2 to 4%, respectively. However, the importance of the aromatic group on the ring nitrogen of the antagonist for antiviral activity is demonstrated by the inactive compounds 16 and 19. By adding a phenyl in 15 or iodophenyl group in 14, 17, 18, and 20 to the structure, the compounds began to show activity at the micromolar level (Fig. 3). On the side of UL141 (site C), negatively charged amino acids Asp232, Glu164, Glu229, Glu230, and Asp228, which are in a direct contact with the antagonist, contribute most significantly to Δ*E*_A:UL_^int^ (Fig. 8). Few nonpolar amino acids as Leu163, Pro231, and Thr235 also contribute significantly. Although Arg85 and Arg208 are also in a direct contact with the antagonist, both the antagonist and the arginines are positively charged, thus, Δ*E*_A:UL-Arg85_^int^ and Δ*E*_A:UL-Arg208_^int^ are repulsive with positive values.

The replacement of the iodine atom (in 14) by a hydrogen atom (in 15) led to an increase of *K*_D_ from 24 μM to 199 μM, indicating the importance of the iodine atom in the structure of the antagonist for antiviral activity. On the other hand, the substitution of the hydroxyl group in the 2-[(*S*)-1-hydroxyethyl] moiety of 14 by a hydrogen atom in 18 demonstrated the importance of less polar and less bulky alkyl group at the position 2 on the pyrrolidine ring of the antagonist compared with the 2-[(*S*)-1-hydroxyethyl] moiety of 14 [*K*_D_(14) = 24 μM decreased to *K*_D_(18) = 2.9 μM]. As can be seen in Figure 7*D*, the bulkier 2-[(*S*)-1-hydroxyethyl] moiety shifted the binding position of the pyrrolidine ring of 14 in site C compared to 18, and the overall interaction energy Δ*E*_A:UL_^int^ of 14 with UL141 as well as Δ*E*_Aring:UL_^int^ [Δ*E*_Aring:UL_^int^ = −163.0 kcal mol^−1^ in 14 *versus* Δ*E*_Aring:UL_^int^ = −182.3 kcal mol^−1^ in 18, Table 2] were weaker. Further decreasing of 2-ethyl to 2-methyl group did not improve potency of the antagonist [*K*_D_(18) = 2.9 μM increased to *K*_D_(20) = 29 μM]. The 2-ethyl group of 18 is located in the upper part of the partially hydrophobic pocket found in site C of the UL141 protein (Fig. 7*B*). The pocket consists of nonpolar amino acid residues on one side (Leu160, Leu163, Ile238) and on the bottom (Ala138, Cys84) and of polar ones on the other side (Arg85, Glu164, and Asp232). Surprisingly, the main contributions to Δ*E*_A:UL_^int^ between the 2-ethyl group of the antagonist 18 and the UL141 protein are from negatively charged amino acid residues (Glu164, Glu229, Asp232, and Asp228, Fig. 7*C*), from which Asp228 and Glu229 are not in direct contact with the 2-ethyl moiety of 18. Of the nonpolar amino acid residues of the hydrophobic pocket, only the interaction with Leu160 is significant. This suggests that the size and chemical nature of the 2-ethyl group is still not optimal for filling the pocket, so further design to improve this structural part of the antagonist is possible.

### Monovalent binding site revealed by mutational analysis

**Figure 9:**
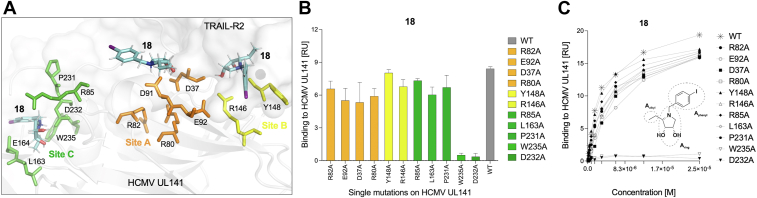
**Mutational analysis of UL141 binding sites for antagonist 18.** Alanine-scanning mutagenesis (*A*) followed by SPR analysis (*B* and *C*) was performed to assess the relative binding requirements for UL141 (*gray* cartoon) and 18 (*cyan sticks*). The mutated residues, shown as sticks in panel (*A*), include Asp91, Glu92, Arg82, Arg80, and Asp37 (site A); Tyr148 and Arg146 (site B); and Arg85, Leu163, Glu164, Pro231, Trp235, and Asp232 (site C). The TRAIL-R2 receptor in transparent surface is shown as seen in the crystal structure (PDB ID: 4I9X). The relative binding responses for compound 18 are presented as the average RU values obtained from three replicates (n = 3) across a concentration range of 0 to 25 μM for each UL141 mutant. These values are plotted in panel (*B*) using the same color coding as in panel (*A*). An alternative representation of the same SPR data, showing all measured concentration points, is provided in panel (*C*), with the structure of compound 18 included for reference.

**Figure 10:**
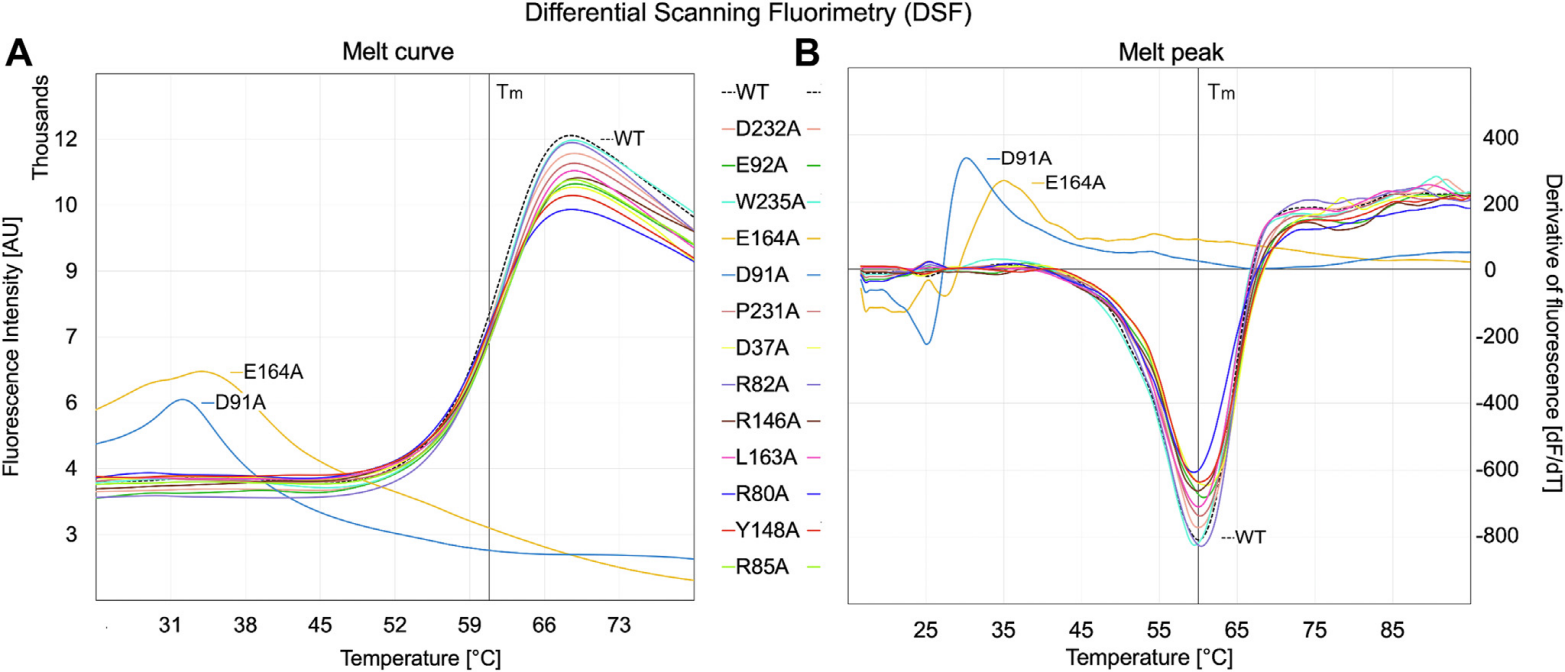
**Differential scanning fluorimetry was conducted to evaluate the relative stability of UL141 mutants in comparison to the WT protein.** The melt curves (*A*) depict the average fluorescence intensity from three replicates (n = 3) as a function of temperature, with measurements recorded at 0.5 °C increments. The melting temperatures (Tm) were determined from the corresponding melt peaks (*B*), represented as the first derivative of fluorescence (dF/dT) plotted against temperature. Data analysis was performed using CFX Manager software (Bio-Rad).

Collectively, the findings from molecular modeling, ELISA, and SPR prompted a more detailed investigation into the binding mechanisms, leading us to explore the possibility of distinct binding sites for UL141 in its interaction with glycomimetic compound 18. To further clarify this, we conducted mutational analysis on the surface residues of UL141 to identify specific binding sites involved in this interaction. The following residues were selected for alanine-scanning mutagenesis, followed by SPR analysis to assess their contribution to the binding affinity of UL141 for 18: Asp91, Glu92, Arg82, Arg80, and Asp37 (site A); Tyr148 and Arg146 (site B); and Arg85, Leu163, Glu164, Pro231, Trp235, and Asp232 (site C) (Fig. 9*A*). The UL141 mutants were generated through single alanine substitutions and recombinantly produced in the BV-Sf9 system following the same protocol as the WT UL141. Alanine substitutions can potentially disrupt the functional integrity of a protein by altering its native composition and structure. In this study, differential scanning fluorimetry identified two mutants, D91A and E164A, that exhibited an early fluorescence increase, suggesting misfolding or aggregation. This aberrant expression is likely due to structural or folding defects introduced by these specific substitutions. In contrast, the remaining mutants displayed a single sigmoidal transition comparable to WT protein, indicative of a properly folded protein, as shown in Figure 10. Consequently, the misfolded mutants were excluded from further analysis to ensure the reliability of downstream experiments. The binding affinities of the remaining mutants were assessed using SPR, as illustrated in Figure 9. Biotinylated UL141 mutants were immobilized on a sensor chip, followed by the injection of compound 18. Binding interactions were monitored over a 300-s period. Notably, certain mutants exhibited no binding to compound 18, suggesting that the antagonist binds to UL141 at a specific site. In conclusion, mutational analysis identified site C as the most likely binding site on UL141, as only the W235A and D232A mutants—key residues within the site C binding interface—prevented binding of the antagonist. This analysis provided deeper insights into the specific interactions governing compound 18 binding, revealing key residues that contribute to its affinity and aligning well with the preceding MM analysis. These findings enhance our understanding of the binding dynamics and mechanisms of inhibition, offering a valuable foundation for the optimization of future pharmacological UL141 antagonists.

## Discussion

Our findings defined the specific direct-acting antagonists of the multifaceted viral glycoprotein HCMV UL141. To our knowledge, these are the first fully synthetic and biocompatible UL141 antagonists, demonstrating potent inhibition of the TRAIL death receptor pathway *in vitro*. The glycomimetic design employed here for the development of UL141 antagonists provides a promising foundation also for further therapeutic advancements. This class of antiviral direct-acting drugs typically targets essential viral components, whereas host-directed therapies focus on disrupting host processes critical for viral replication (26). Insightfully, the iminosugars (subclass of glycomimetics) seem to be promising candidates for such developments (27). Their antiviral activity as host-directed drugs have been proved against a range of enveloped viruses, including dengue virus (28, 29), influenza virus (30, 31), hepatitis C virus (32, 33), human immunodeficiency virus (34, 35), as well as severe acute respiratory syndrome coronavirus 2 (36, 37). Antiviral activity of iminosugars is also related to their ability of inhibiting the glycosidases, the enzymes which are involved in the biosynthesis and processing of *N*-glycans. Inhibition of glycosidases has profound effect on the glycan structure, and consequently, affects function of glycoproteins, and could therefore alter processes as cell-cell and cell-virus recognition (48). In the last decade, we have synthesized a library of five-membered iminosugars, *N*-substituted 1,4-dideoxy-1,4-imino-L-lyxitols (44, 45, 46, 47) and 1,4-dideoxy-1,4-imino-D-lyxitols (49, 50) and tested their inhibitory activity against various cellular targets (*e.g.*, Golgi or lysosomal α-mannosidases II) with the overall aim to develop an inhibitor with anticancer and/or antiviral activity.

This established link between glycosidase inhibition and antiviral activity was harnessed, enabling here the successful screening of selected glycomimetics against this viral protein to develop a UL141 antagonist as a direct-acting drug. The structure of the identified lead compound was subsequently refined to enhance its potency as an antagonist and to clarify the binding mechanism between the iminosugar and the viral protein. Utilizing the crystal structures and molecular modeling, the binding site of the newly synthesized antagonist on the surface of UL141 was proposed and confirmed through mutational analysis. Surprisingly, the primary contributions to the interaction between the antagonist 18 and the UL141 protein arise from negatively charged residues (Glu164, Glu229, Asp232, and Asp228), despite Asp228 and Glu229 not being in direct contact with the 2-ethyl moiety of the antagonist. The limited interaction with nonpolar residues, particularly Leu160, indicates that the size and chemical properties of the 2-ethyl group are suboptimal, leaving room for further structural refinement of the UL141 antagonist for direct inhibition of the specific protein–protein interaction (PPI) *in vitro*. The PPI inhibitors represent a transformative groundbreaking advancement in drug discovery, targeting critical cellular interactions implicated in various diseases, including cancer, infectious diseases, and neurodegenerative disorders (51, 52, 53, 54, 55, 56). These inhibitors aim to disrupt PPIs by targeting “hot spots”—specific regions within protein interfaces that are essential for interaction stability and specificity. However, the design of effective PPI inhibitors remains a significant challenge due to the typically large, flat, and dynamic nature of protein interfaces (such as UL141), which often lack the well-defined binding pockets characteristic of enzyme targets (57). To overcome these challenges, researchers have employed a range of strategies, including the development of small molecules (57), peptides (54), and macrocyclic compounds (58). Small molecules, for instance, can restore tumor suppressor activity by preventing protein degradation and have established a paradigm for targeting intracellular pathways in cancer (57, 59, 60). Peptides, particularly those derived from native interaction motifs and stabilized through techniques like hydrocarbon stapling (61, 62), effectively mimic secondary structural elements, enhancing binding specificity and bioavailability. Macrocyclic inhibitors, which integrate the advantageous properties of both peptides and small molecules, offer superior stability and target selectivity (58). Advances in fragment-based drug discovery and high-throughput screening have further facilitated the identification of novel scaffolds capable of disrupting PPIs with high precision. These approaches underscore the immense potential of PPI inhibitors, including glycomimetics, in addressing diseases traditionally considered “undruggable” by conventional therapies.

Moreover, the combined studies oriented on viral immunoevasins offer profound insights that extend beyond understanding viral–host interactions and offering substantial potential to drive advancements in drug discovery (1, 22, 39, 63). For example, the unique ability of UL141's capacity to circumvent immune defenses, combined with its role as a critical priming component of HCMV, has been identified as a defining hallmark in cancer biology (39), highlighting the emerging parallels between viral immune evasion and tumor immune suppression. Exploring viral strategies for immune modulation could provide a foundation for innovative approaches in cancer immunotherapy. Notably, several viral immunoevasins, initially identified as immune escape factors, have recently been implicated in antitumor immunity (64, 65). In addition, the computational approaches, such as molecular docking and molecular dynamics simulations, are instrumental in elucidating interaction mechanisms and optimizing inhibitor design. Despite significant advancements, challenges persist in the field, including issues with low solubility, cytotoxicity, limited bioavailability, and the complexity of inhibitor synthesis. Addressing these limitations requires a multidisciplinary approach that integrates structural and cellular biology, synthetic chemistry, and pharmacology.

## Conclusion

UL141 was previously recognized primarily for its intracellular role in immune evasion, where it restricts the expression of immune molecules on the surface of infected cells through novel, noncanonical interactions with death receptors and employs its versatile immunoglobulin-like fold to modulate immune checkpoints *via* binding to CD155 (17, 18, 19, 20, 21). Recent studies, however, have revealed additional, distinct, and critical function of UL141 in the HCMV life cycle (1). Specifically, its association with the gH/UL116 complex in the virion envelope enhances viral infectivity, suggesting that UL141 serves as a receptor-binding moiety that facilitates HCMV spread. To target this multifaceted role of UL141, we developed and characterized specific direct-acting antagonists *in vitro*. To our knowledge, these are the first fully synthetic and biocompatible HCMV UL141 antagonists, demonstrating potent inhibition of the death receptor (TRAIL-R2) interaction at the micromolar scale. This glycomimetic design represents a promising foundation for the further development of UL141-targeted therapeutics.

## Experimental procedures

### Synthesis of novel compounds

All commercially available reagents and anhydrous solvents were used as received. Specific optical rotations were determined on a Jasco P-2000 polarimeter at 20 °C. ^1^H NMR and ^13^C NMR spectra were recorded at 25 °C with a Bruker AVANCE III HD 400 spectrometer. Chemical shifts are given in ppm (*δ*) relative to residual signal of appropriate deuterated solvent used (CD_3_OD). Coupling constants are given in Hz. All given ^13^C spectra are proton decoupled. High resolution mass spectra were obtained using Orbitrap Elite (Thermo Fisher Scientific) mass spectrometer with ESI ionization in positive mode. Thin-layer chromatography was performed on alumina sheets precoated with thin-layer chromatography Silica gel 60 F254 (Merck). Visualization was achieved by immersing the plates into PMA solution (10% solution of phosphomolybdic acid in ethanol) and heating at ca 200 °C with a heat gun. Column chromatography was performed as flash chromatography on Silica gel 60 (0.040–0.063 mm, Merck). Solvents used for flash chromatography were of technical grade and distilled before use. The compounds for biological assays were lyophilized before the use. The structure of all derivatives was confirmed by NMR spectroscopy.

#### (2S,3R,4S)-2-((S)-1-hydroxyethyl)pyrrolidine-3,4-diol hydrochloride (16)

The catalyst was filtered, the filtrate was cooled to 0 °C and acidified with conc. HCl. Then, the solvent was evaporated, and the product was freeze-dried to afford 16 as brownish crystals (27 mg, 94%). [α]_D_ = + 31.7 (*c* = 0.25, H_2_O). ^1^H NMR (400 MHz, CD_3_OD): δ 4.31 (td, *J* = 8.3, 3.7 Hz, 1H, H-4), 4.19 (dd, *J* = 3.7, 3.0 Hz, 1H, H-3), 4.07 (dq, *J* = 8.1, 6.4 Hz, 1H, C*H*OH), 3.39 (dd, *J* = 11.6, 8.3 Hz, 1H, H-5), 3.04 (dd, *J* = 11.6, 8.4 Hz, 1H, H-5′), 3.22 (dd, *J* = 8.1, 3.0 Hz, 1H, H-2), 1.22 (d, *J* = 6.4 Hz, 3H, CH_3_) ppm. ^13^C NMR (101 MHz, CD_3_OD): δ 71.8 (C-4), 71.2 (C-3), 67.7 (C-2), 64.6 (CHOH), 48.7 (C-5), 20.7 (CH_3_) ppm. HRMS (ESI-MS): *m/z*: calcd for C_6_H_13_NO_3_ [M + H]^+^: 148.0968, found: 148.0966.

#### (2S,3R,4S)-2-ethylpyrrolidine-3,4-diol hydrochloride (19)

A suspension of 19a (45) (312 mg, 1.2 mmol) and 10% Pd-C (31.2 mg, 10% wt) in MeOH (10 ml) was stirred under H_2_ atmosphere (balloon) at RT for 12 h. The catalyst was filtered, the filtrate was cooled to 0 °C, and acidified with conc. HCl. Then, the reaction mixture was stirred at 40 °C for 2 h. After cooling to RT, the solvent was evaporated, and the product was freeze-dried to afford 19 as brownish crystals (158 mg, 79%). [α]_D_^20^ = +13.5 (*c* = 0.25, CH_3_OH). ^1^H NMR (400 MHz, CD_3_OD): δ 4.43 (td, 1H, *J* = 8.1, 3.8 Hz, H-4), 4.12 (t, 1H, *J* = 3.5 Hz, H-3), 3.50-3.39 (m, 2H, H-5, H-2), 3.10 (dd, 1H, *J* = 11.6, 8.1 Hz, H-5′), 2.01-1.87 (m, 1H, C*H*_*2*_CH_3_), 1.85-1.72 (m, 1H, C*H*_*2*_CH_3_), 1.07 (t, 3H, *J* = 7.5 Hz, CH_2_C*H*_*3*_) ppm. ^13^C NMR (101 MHz, CD_3_OD): δ 72.0 (C-4), 71.3 (C-3), 65.2 (C-2), 48.4 (C-5), 21.0 (*C*H_2_CH_3_), 10.9 (CH_2_*C*H_3_) ppm. HRMS (ESI-MS): *m/z*: calcd for C_6_H_13_NO_2_ [M + H]^+^: 132.1019, found: 132.1027.

#### (2S,3R,4S)-2-methylpyrrolidine-3,4-diol hydrochloride (21)

A suspension of 21a (45) (0.18 g, 0.7 mmol) and 10% Pd/C in MeOH was stirred under H_2_ atmosphere (balloon) at RT for 4 h. The catalyst was filtered, the filtrate was cooled at 0 °C, and acidified with conc. HCl. Then, the mixture was stirred at 40 °C for 2 h. After cooling to RT, the solvent was evaporated and freeze-dried to afford 21 as yellowish solid (73 mg, 66%). [α]_D_ = + 30.8 (*c* = 0.25, CH_3_OH). ^1^H NMR (400 MHz, CD_3_OD): δ 4.44 (td, 1H, *J* = 7.7, 4.0 Hz, H-4), 4.05 (t, 1H, *J* = 3.7 Hz, H-3), 3.64 (qd, 1H, *J* = 6.7, 3.2 Hz, H-2), 3.46 (dd, 1H, *J* = 11.7, 7.9 Hz, H-5), 3.11 (dd, 1H, *J* = 11.8, 7.4 Hz, H-5′), 1.43 (d, 3H, *J* = 6.8 Hz, CH_3_) ppm. ^13^C NMR (101 MHz, CD_3_OD): δ 72.6 (C-3), 72.1 (C-4), 59.2 (C-2), 49.0 (C-5), 12.2 (CH_3_) ppm. HRMS (ESI-MS): *m/z*: calcd for C_5_H_11_NO_2_ [M+H]^+^: 118.0863, found: 118.0878.

### Cell source and maintenance

#### Cell lines

The human dermal fibroblast cell line HFF-BJ (CRL-2522) was procured from the American Type Culture Collection (ATCC). HFF-BJ cells, derived from neonatal foreskin tissue, are widely recognized as a mammalian fibroblast model extensively used in biomedical research. They are particularly valuable for replicating and isolating the HCMV, making them a standard model for virological studies. Additionally, this cell line is frequently employed in drug screening assays, offering a reliable platform for testing therapeutic compounds. Other cell systems used in this study included *Escherichia coli* XL10-Gold and *E. coli* JM109 strains, employed for propagating expression DNA plasmids, and *Spodoptera frugiperda*-derived Sf9 insect cells, utilized for the production of recombinant proteins. The bacterial strains were obtained from Agilent Technologies, while the Sf9 cells were procured from Expression Systems.

#### Cell culture maintenance

The HFF-BJ cells were cultured in Dulbecco's Modifed Eagle Medium/Nutrient Mixture F-12 media (DMEM/F-12) supplemented with 10% heat-inactivated fetal bovine serum, 1% antibiotics (100 unit/ml of penicillin and 100 μg/ml of streptomycin), and 1 mM sodium pyruvate. All cell culture reagents were purchased from Thermo Fisher Scientific. The cell lines were maintained in a humidified incubator at 37 °C with 5% CO_2_. Cells were subcultured regularly three times a week, keeping them in the exponential growth phase. All cell lines are regularly tested by PCR for common species of *mycoplasma* by using primers: 5′-ACACCATGGGAGYTGGTAAT-3′ (forward), 5′-CTTWTCGACTTYCAGACCCAAGGCAT-3′ (reverse); and nested PCR primers: 5′-GTGSGGMTGGATCACCTCCT-3′ (forward), 5′-GCATCCACCAWAWACYCTT-3′ (reverse) (66, 67).

### Compounds stocks

Individual compound solutions were prepared by dissolving the solid compounds in sterile DMSO and filtering the resulting solution through a 0.2 μm syringe filter. The stock solutions were standardized to a concentration of 10 mg/ml, equivalent to approximately 25 to 65 mM, depending on the molecular weight (Mw) of each compound. These stock solutions, containing 10 to 25% DMSO, were further diluted to 1 mM in PBS. To preserve their stability, all stock solutions were stored at −20 °C under light-protected conditions. For experimental use, the stock solutions were diluted to working concentrations ranging from 0.1 to 30 μM in cell culture medium for cytotoxicity assays and ranging from 0.005 to 530 μM in the appropriate buffers for binding assays. Based on prior experience, it was observed that high DMSO concentrations (>0.6%) compromise the reproducibility of binding assay results. Therefore, the final working concentrations of DMSO in all samples were carefully minimized (maintained at a maximum of 0.03–0.5%, respectively) to ensure reliable data acquisition. This adjustment also facilitates the accurate assessment of PPIs for low-affinity small-molecule inhibitors, which are often solubilized in DMSO.

### Cytotoxicity and cell viability

Cells were seeded in a 96-well plate at a density of 1 × 10^4^ cells per well for the measurement of cytotoxicity of the compounds (without UVA irradiation). Following 24 h of incubation, the cells were treated with various concentrations ranging from 0.1 to 30 μM of test compounds (1–21) and followed by microscopy inspection. Cells after treatment were incubated for 48 h. Untreated cells were considered negative control, and DMSO-treated cells (at appropriate concentration) were considered vehicle control. The classical MTT assay (3-(4,5-dimethyl thiazolyl-2)-2,5-diphenyltetrazolium bromide) (68) was used to measure cellular metabolic activity as an indicator of cell viability, proliferation, and cytotoxicity. Briefly, 0.5 mg/ml MTT solution was dispensed in each well of the 96-well plate and incubated for 4 h. Afterward, the MTT solution was removed and 200 μl of 100% DMSO was added to each well and the plate was placed on a microplate shaker for 5 min to dissolve the formazan granules. The absorbance was measured at 550 nm using a spectrophotometer. DMSO and MTT were purchased from Thermo Fisher Scientific. The darker the solution, the greater the number of viable, metabolically active cells. All experimental compounds were measured using at least 2 to 3 replicates for each and the averaged values from three independent experiments (n = 3) are presented. Cell viability was visualized by fold change of formazan-positive cells normalized to the untreated control and DMSO. Viability reduced below 40% represents the cytotoxic zone. All evaluations were performed using GraphPad Prism 9.5.0 (GraphPad Software, LLC), if other software was not explicitly mentioned.

### Cloning, expression, and purification of recombinant proteins

#### Generation of expression constructs

The generation of expression plasmids followed established protocols with minor modifications as outlined in previous studies (17, 18). DNA encoding the extracellular domain of HCMV UL141 (residues 30–300, Gene ID: 3077418) was amplified *via* PCR using an HCMV BAC clone as the template (19, 21). The amplified fragment was subsequently inserted downstream of the gp67 signal sequence within the baculovirus transfer vector pAcGP67A (BD Biosciences). Constructs were generated with or without the addition of an N-terminal octa-histidine tag or a C-terminal human IgG_1_(Fc) fusion domain for enhanced expression and purification. Additionally, the ectodomain of human TRAIL-R2 (amino acids 58–184, Gene ID: 8795) was expressed as an Fc-fusion protein. This construct was generated following a previously described methodology in Nemčovičová *et al.*, 2013 (17). These expression systems were optimized to ensure efficient production of the recombinant proteins for subsequent biochemical and structural analyses.

#### Generation of mutants

Specific point mutants of HCMV UL141, including D91A, E92A, R82A, R80A, D37A, Y148A, R146A, R85A, L163A, E164A, P231A, W235A, and D232A (numbering starting from initiating methionine), were generated within the same expression vector. Mutagenesis was performed using complementary pairs of single-stranded mutagenic primers (oligonucleotides) and the QuikChange II Site-directed mutagenesis Kit (Agilent). The accuracy of the inserted gene sequences and the successful incorporation of mutations were confirmed *via* DNA sequencing. Following sequence verification, the plasmids were amplified in *E. coli* strains (XL10-Gold or JM109) using the EndoFree Plasmid Maxi Kit (Qiagen). The plasmids were maintained under sterile conditions to ensure integrity and suitability for subsequent applications. This workflow ensured high fidelity and consistency in the generation of mutant constructs for experimental use.

#### Protein expression and purification

HCMV glycoprotein UL141 (variants UL141FL-8H, UL141FL, UL141FL-Fc, and UL141 mutants) and the human TRAIL-R2 receptor (TRAIL-R2-Fc) were expressed using a baculovirus-mediated expression system in *S. frugiperda* (Sf9) cells. The detailed protocol for recombinant baculovirus preparation was recently described by Bitala *et al.*, 2025 (69), while protein isolation and purification procedures were adapted from Nemčovičová *et al.*, 2013 (18). Following a 72-h expression period in insect cell media, the culture supernatant containing the target protein was clarified by centrifugation to remove cells and debris. The clarified supernatant was concentrated and buffer-exchanged into 50 mM Tris/HCl, pH 8.0, 300 mM NaCl using tangential flow filtration. Affinity chromatography was performed as the primary purification step, followed by ion-exchange chromatography for further purification. Protein-containing fractions were dialyzed to remove impurities such as imidazole or free biotin, pooled, and concentrated before being loaded onto a Superdex S75 size-exclusion chromatography column (Cytiva). SDS-PAGE analysis confirmed protein purity, with fractions achieving greater than 95% purity pooled for final use. Proteins were concentrated in a buffer suitable for downstream applications, typically 50 mM Hepes, pH 7.5, 150 mM NaCl. The final protein concentration and yield were determined spectrophotometrically using a NanoDrop instrument (Thermo Fisher Scientific), ensuring consistency and reliability of the preparations for subsequent experimental assays.

### Binding assays

#### Biotinylation of UL141

To prepare HCMV UL141 for binding studies, biotinylation was performed using the BiotinTag Micro Biotinylation Kit (Sigma-Aldrich). Purified UL141 protein (50 μg in 50 μl) was mixed with 50 μl of BiotinTag reaction buffer provided in the kit. Sulfo-NHS-Biotin reagent was freshly prepared by dissolving the vial contents in 1 ml of sterile water. A calculated volume of the biotin reagent was added to achieve a 20:1 M excess of biotin to protein. The reaction mixture was incubated for 30 min at room temperature with gentle mixing to ensure efficient labeling. Following incubation, unreacted biotin was removed using the desalting column included in the kit. The biotinylated UL141 protein was eluted in 50 mM Hepes, pH 7.5, 150 mM NaCl. Biotin incorporation was verified using a colorimetric biotin quantification assay (*e.g.*, HABA assay). The biotinylated protein was then quantified spectrophotometrically using a NanoDrop instrument and concentrated to the desired level for Biacore or ELISA binding studies. The biotinylated protein was stored at 4 °C for short-term use or −80 °C for long-term storage to maintain stability.

#### ELISA binding assay

The binding of recombinant HCMV UL141 to human TRAIL-R2 and selected glycomimetics was evaluated using an ELISA-TMB assay. Biotinylated UL141 protein (2 μg) was immobilized onto Dynabeads M-280 Streptavidin, which were resuspended and washed three times with PBS-T (PBS + 0.05% Tween-20). Protein–bead complexes were incubated at room temperature for 30 min with gentle rotation, washed with PBS to remove unbound protein, and 50 μl of the bead suspension was added to each well of a 96-well plate, stabilized with PBS, and stored at 4 °C. Next, glycomimetics (1–21, 25 μM) or a buffer control (containing appropriate DMSO) were added and incubated for 15 min at room temperature and washed, followed by TRAIL-R2 (2 μg) incubation for 15 min. Plates were washed twice with PBS-T and incubated with HRP-conjugated antibody (1:2000, Thermo Fisher Scientific) targeting the control protein's Fc region. After washing, plates were developed for 5 to 10 min with 50 μl/well soluble TMB substrate (Sigma-Aldrich), and the reaction was stopped with 50 μl/well TMB Stop solution. Data were performed in two to three replicates. The step-by-step procedure is presented in Figure 4*A*. Absorbance at 450 nm was measured, and data (mean ± SD) from three independent experiments (n = 3) were analyzed using GraphPad Prism 9.5.0 to differentiate bound from unbound TRAIL-R2.

#### SPR-binding assay

SPR experiments were conducted using a BIACORE 3000 instrument (Cytiva), following established protocol for investigating UL141 interaction with TRAIL-R2 and CD155 (17, 18, 19). The sensor surface of an SA chip (Cytiva) was preconditioned with HBS-EP buffer (10 mM Hepes, 150 mM NaCl, 3 mM EDTA, 0.005% Tween-20) to stabilize the baseline and remove any loosely bound streptavidin. Before use, the buffer was filtered through a 0.2 μm polystyrene filter and degassed under vacuum for 10 min to eliminate air bubbles. Biotinylated HCMV UL141 was immobilized on the SA chip, maintaining biotinylation at 0.5 to 1.5 mol per mole of protein. Excess biotin was removed *via* gel filtration. UL141 was injected at 30 μl/min, achieving maximum immobilization (4000–6000 RU), followed by washing with running buffer. Residual unbound proteins were optionally removed with a 2 M NaCl pulse. To ensure optimal SPR signal detection, which depends on the analyte mass bound to the ligand, relatively high amounts of ligand were immobilized on the sensor chip to achieve a significant response signal upon binding of the small compound. Analytes, including glycomimetics (0–530 μM) and TRAIL-R2 (0–0.37 μM), were injected at 50 μl/min at 25 °C in HBS-EP buffer for 7.5 min, followed by a 15-min dissociation phase. Regeneration of the chip surface, when necessary, was performed using 5 mM NaOH. Control injections of zero concentrations and duplicate injections of selected concentrations were included to validate surface regeneration. Data were analyzed by regression analysis and fitted to a four-parameter logistic sigmoidal dose-response curve using GraphPad Prism 9.5.0 (GraphPad Software, LLC). The goodness of fit was evaluated based on R^2^ values (typically 0.80–0.98), and kinetic and affinity parameters were derived from processed data obtained from two to three independent experiments.

#### Differential scanning fluorimetry

Fluorescence thermal shift assays were conducted to assess the thermal stability of WT UL141 and its mutants (D91A, E92A, R82A, R80A, D37A, Y148A, R146A, R85A, L163A, E164A, P231A, W235A, and D232A). A protein/dye solution was prepared by mixing 420 μl of each UL141 variant at a concentration of 0.5 to 2 mg/ml with 2.1 μl of 5000 × SYPRO Orange dye (Sigma-Aldrich), followed by dilution in 50 mM Hepes, pH 7.5, 150 mM NaCl to a final volume of 525 μl. Using a multichannel pipette, 20 μl of the prepared buffer was transferred into a PCR plate, which was then centrifuged at 1500*g* for 30 s. Subsequently, 5 μl of the protein/dye solution was added to each well. The plate was sealed, gently tapped to ensure proper mixing, and centrifuged again under the same conditions. The thermal shift assay was performed using a real-time PCR machine (Bio-Rad) with a temperature gradient ranging from 15 °C to 95 °C, increasing in 0.5 °C increments, while fluorescence intensity was recorded at each step. Data analysis was carried out using CFX Manager software (Bio-Rad) to determine the melting temperature (Tm) of each UL141 variant, providing insights into their relative stability. All DSF measurements were performed in duplicates.

### Molecular modeling

#### Docking with Glide

The X-ray structures of human cytomegalovirus glycoprotein UL141 with the death receptor TRAIL-R2 (PDB ID: 4I9X) (17, 18) was used as a 3-D protein model for molecular docking of synthetic antagonists using the GLIDE program (70, 71) of the Schrödinger package. The protein:antagonist complexes consisted of UL141 and the compounds 14 and 18 were built. Protonation forms of amino acid residues of UL141 and amino groups of the antagonists were calculated for the pH = 7.0 using the Propka v.2 program (72, 73). The synthesized compounds were docked on a surface of UL141 which is a part of the binding interface between UL141 and TRAIL-R2. This surface was divided into three docking grids, the first one centered around Arg82 amino acid residue, the second one centered around Tyr148, and third one centered around Trp235, based on important interactions between UL141 and TRAIL-R2 described in an X-ray structure of the protein complex (17) and previous theoretical FMO-PIEDA calculations (74). The receptor box for the docking conformational search was centered at three sites (around Arg82, Trp235, and Tyr148 amino acid residues) with a size of 39 × 39 × 39 Å using partial atomic charges for the receptor from the OPLS2005 force field (75). For antagonists, the charges were calculated at the quantum mechanics level with the density functional theory method (M06-2X/LACVP∗∗) (76, 77) using the Jaguar program (78) of the Schrödinger package. The grid maps were created with no Van der Waals radius and charge scaling for the atoms of the receptor. Flexible docking in standard and extra precision was used. All antagonists were docked with the amino group at the pyrrolidine ring in a protonated form according to Propka calculations. The potential for nonpolar parts of the ligand was softened by scaling the Van der Waals radii by a factor of 0.8 for atoms of the ligands with partial atomic charges less than specified cut-off of 0.15. The postdocking minimization for 10 ligand poses with the best docking score was performed and optimized structures were saved for subsequent analyses using the MAESTRO viewer (79) of the Schrödinger package.

#### QM/MM geometry optimizations

Geometries of selected complexes (antagonist 14:UL141 monomer and antagonist 18:UL141 monomer) from molecular docking were subsequently optimized at the QM/MM levels (BP86/LACVP∗:OPLS2005 and M06-2X/LACVP∗:OPLS2005) (75, 77, 80, 81) using the QSite (82, 83) program of the Schrödinger package. For each complex, two docking poses of the antagonist 14 as well as the antagonist 18 were selected for the subsequent QM/MM geometry optimizations. The following decomposed scheme was used: the QM part consists of the antagonist and amino acid residues of UL141 in direct contact with the bound antagonist. The rest of the protein was included into the MM part and described by the OPLS2005 force field (75). The QM/MM methodology (an additive scheme) with hydrogen caps on boundary QM atoms and electrostatic treatment at the interface between the QM and MM regions using Gaussian charge distributions represented on a grid was employed.

#### pK_a_ calculations with Propka

The complexes of the docked antagonists with the UL141 protein were used to predict p*K*_a_ values and preferred ionizable forms of amino acid residues of the protein (for pH = 7) as well as a bound antagonist using the empirical method, the Propka v.2. (72, 73).

#### FMO-PIEDA calculations

PIEDA was used along the two-body FMO method (84, 85, 86). In FMO method, a biomolecular system is partitioned into fragments (with a fragment size – one amino acid residue). For modeling of the covalently bounded amino acids, the hybrid orbital projection operator technique was applied. Inputs for FMO-PIEDA calculations were built from the QM/MM optimized complexes using the Facio program (87). The FMO calculations were performed using the second-order Møller-Plesset theory (88, 89) (MP2) with the 6-31G(d) basis and conductor-like polarizable continuum model (C-PCM, PCM<1>, keywords IFMO = −1, IEF = −10, SOLVNT = WATER) (90). The Gamess package (91, 92) [version 30 June 2021 (R1)] was used. The virtue of the FMO technique is to predict pair interactions between the two structural fragments of the molecular system embedded within the electrostatic potential of the surroundings (IFIE, inter fragment interaction energy). This method was recently used to analyze interaction energy in complexes of inhibitors with α-mannosidases from the family GH38 (49, 50, 93) as well as for complexes of α_IIb_β_3_ integrin with peptidic and nonpeptidic ligands (94).

To understand a binding effect of structural moieties of the antagonists (A) 14 and 18, they were divided into three fragments, the pyrrolidine moiety (Aring), iodophenyl moiety (Aphenyl), and etyl (in the case of 18 or hydroxyethyl in the case of 14) moiety (Aalkyl) (Fig. 7*E*). Then, the total pair interaction energy between the antagonist and the UL141 protein (Δ*E*_A:UL_^int^) consists of the pair interaction energy between the pyrrolidine ring of the antagonist and the UL141 protein (Δ*E*_Aring:UL_^int^), the pair interaction energy between the iodophenyl moiety of the antagonist and the UL141 protein (Δ*E*_Aphenyl:UL_^int^), and the pair interaction energy between the ethyl moiety (in case of the structure 18) or hydroxyethyl moiety (in case of the structure 14) of the antagonist and the UL141 protein (Δ*E*_Aalkyl:UL_) (Equation 1):

(1) $$\Delta E_{{A:\text{UL}}^{\mathrm{int}}}=\Delta E_{{\text{Aring}:\text{UL}}^{\mathrm{int}}}+\Delta E_{{\text{Aphenyl}:\text{UL}}^{\mathrm{int}}}+\Delta E_{{\text{Aalkyl}:\text{UL}}^{\mathrm{int}}}$$

## Data availability

All research data are contained within the manuscript. Further information and requests for resources, data, and reagents should be directed to and will be fulfilled by the Lead Contact, Ivana Nemčovičová (viruivka@savba.sk). Cells, constructs, or special reagents are available upon request, subject to our institutional and material transfer agreements.

## Supporting information

This article contains supporting information.

## Conflict of interest

The authors declare that they have no conflicts of interest with the contents of this article.
