# Supplementary material for: Small glycomimetic antagonists of the cytomegalovirus glycoprotein UL141 prevent binding to TRAIL death receptor
Source: J Biol Chem. 2025 Apr 10;301(5):108490. doi: 10.1016/j.jbc.2025.108490 (PMC12140054; doi:10.1016/j.jbc.2025.108490)

**Supporting information**

**Small glycomimetic antagonists of the cytomegalovirus glycoprotein UL141**

**prevent binding to TRAIL death receptor**

*Ivana Nemčovičová^1,*^, Juraj Kóňa^2,3^, Monika Poláková^2^, Tomáš Klunda^2^, Andrej Bitala^1^, Mário Benko^1^, Simona Lenhartová^1,#^, Marek Nemčovič^2^*

*^1^ Biomedical Research Center (BMC), Slovak Academy of Sciences, Bratislava, Slovakia*

*^2^ Institute of Chemistry, Slovak Academy of Sciences, Bratislava, Slovakia*

*^3^ Medical Vision, o. z., Bratislava, Slovakia*

**Content**

Figure S1 page 2

Figure S2 page 3

Table S1 page 4

^1^H and ^13^C NMR spectra of the compounds **16**, **19** and **21** pages 5-7

*^*^To whom correspondence should be addressed: Dr. Ivana Nemčovičová, Biomedical Research Center, Dúbravská cesta 9, 845 05 Bratislava, Slovakia, Telephone: (+421)-2-59302-435; E-mail:* [*viruivka@savba.sk*](mailto:viruivka@savba.sk)

*^#^Present address: F. D. Roosevelt University Hospital, Department of Clinical Hematology, Banská Bystrica, Slovakia*


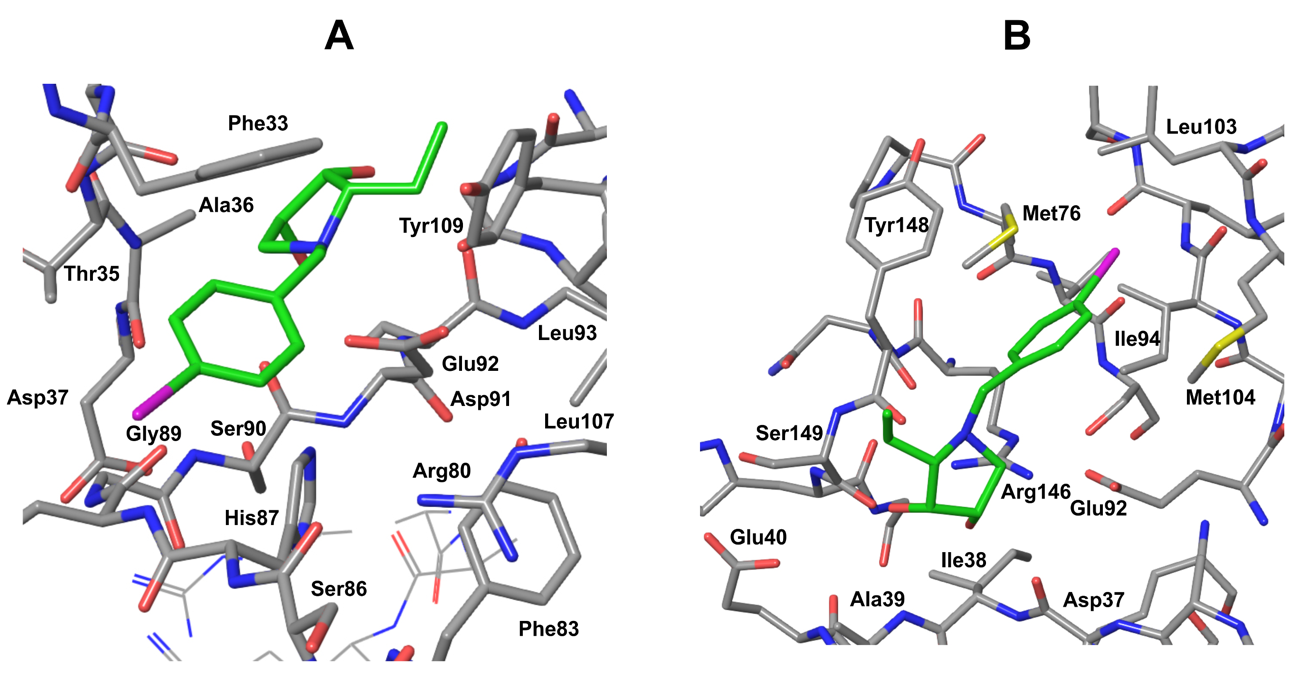


**Figure S1.** The antagonist 18 bound at Site A (A) and Site B (B), hydrogens are not visualized for clarity.


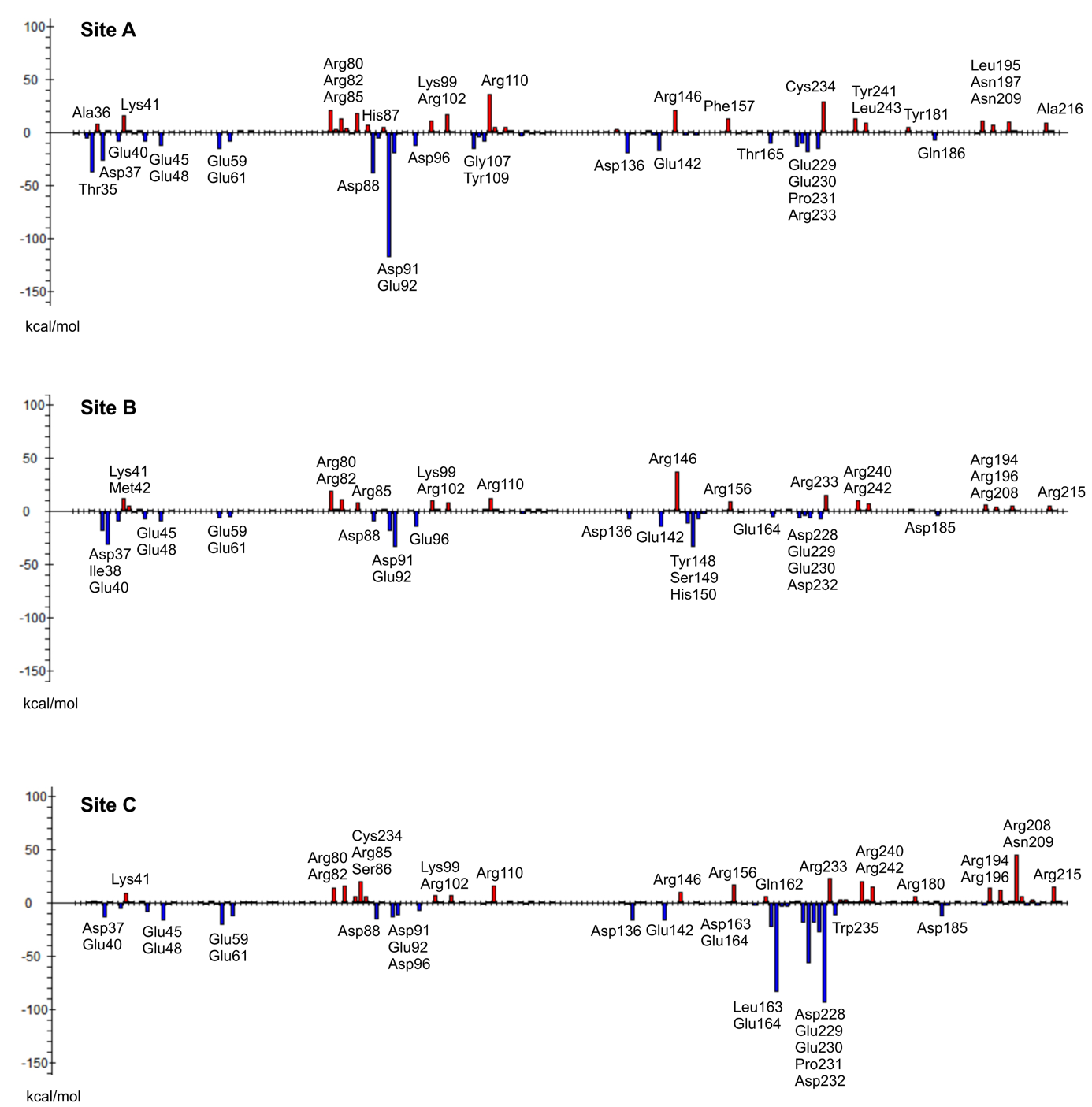


**Figure S2.** FMO pair interaction energies (ΔE_A:UL_^int^) (in kcal mol^-1^) between the antagonist 14 and the UL141 protein (biding sites A, B and C). The most significant ΔE_A:UL_^int^ are marked.

**Table S1.** Total interaction energy (ΔE_A:UL_^int^) (in kcal mol^−1^) between the antagonists 14, 18 and UL141 protein (Sites A, B and C) calculated with the FMO-PIEDA method at the MP2//BP86 level. The pyrrolidine ring conformations of the bound antagonists are also compiled.

| **Structure 14** | **binding close to** | **conform** | **ΔE_A:UL_^int^** |
| --- | --- | --- | --- |
| Site A (pose 1) | Asp91 | E_3_ | -164.8 |
| Site B (pose 1) | Tyr148 | E_5_/^4^E | -115.6 |
| Site B (pose 2) | Tyr148 | ^1^E | -98.6 |
| Site C (pose 1) | Trp235 | E_5_ | -225.6 |
| Site C (pose 2) | Trp235 | ^4^E | -182.2 |
| **Structure 18** |  |  |  |
| Site A (pose 1) | Asp91 | E_5_ | -181.0 |
| Site B (pose 1) | Tyr148 | E_2_/^1^E | -97.5 |
| Site B (pose 2) | Tyr148 | E_2_/^1^E | -96.3 |
| Site C (pose 1) | Trp235 | ^4^E | -220.5 |
| Site C (pose 2) | Trp235 | E_5_ | -207.5 |


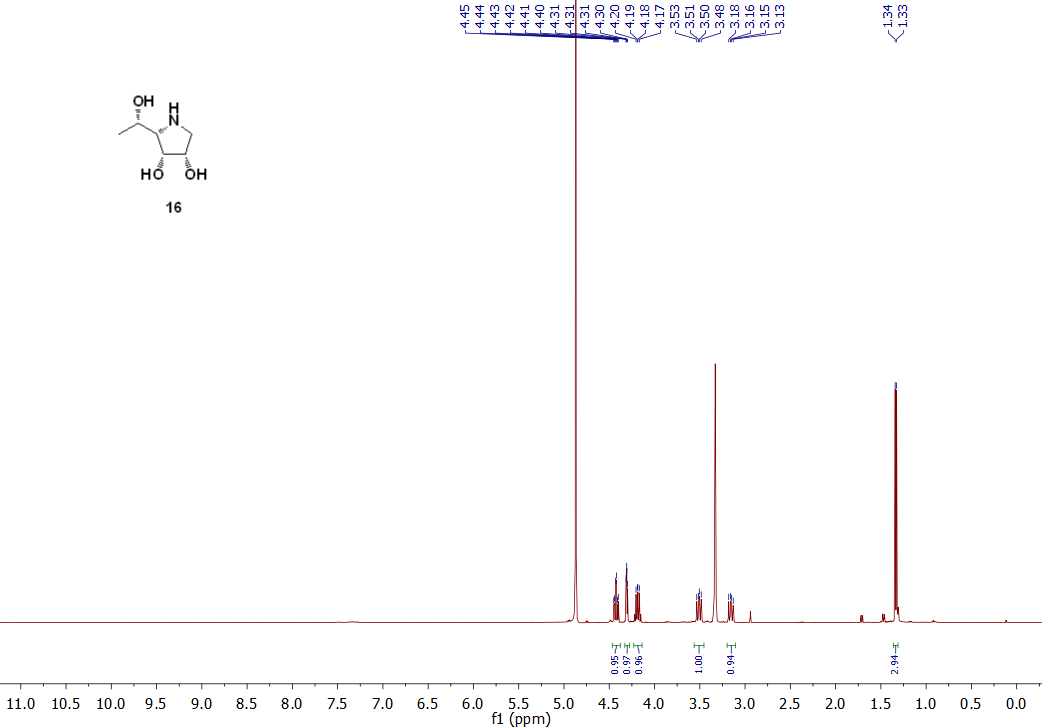


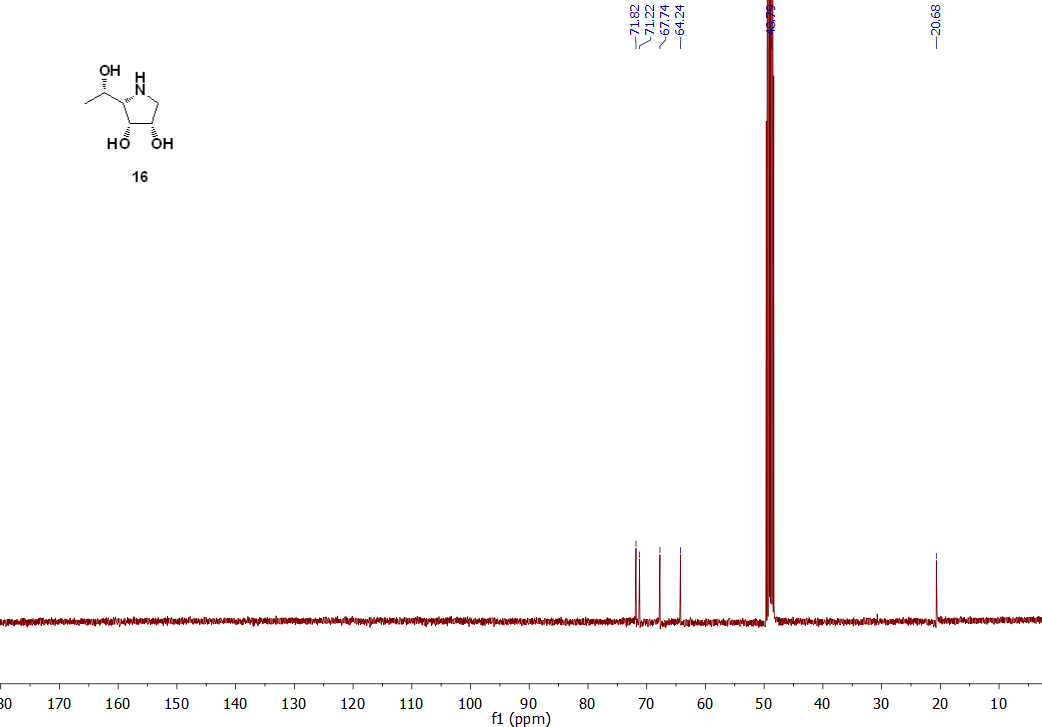


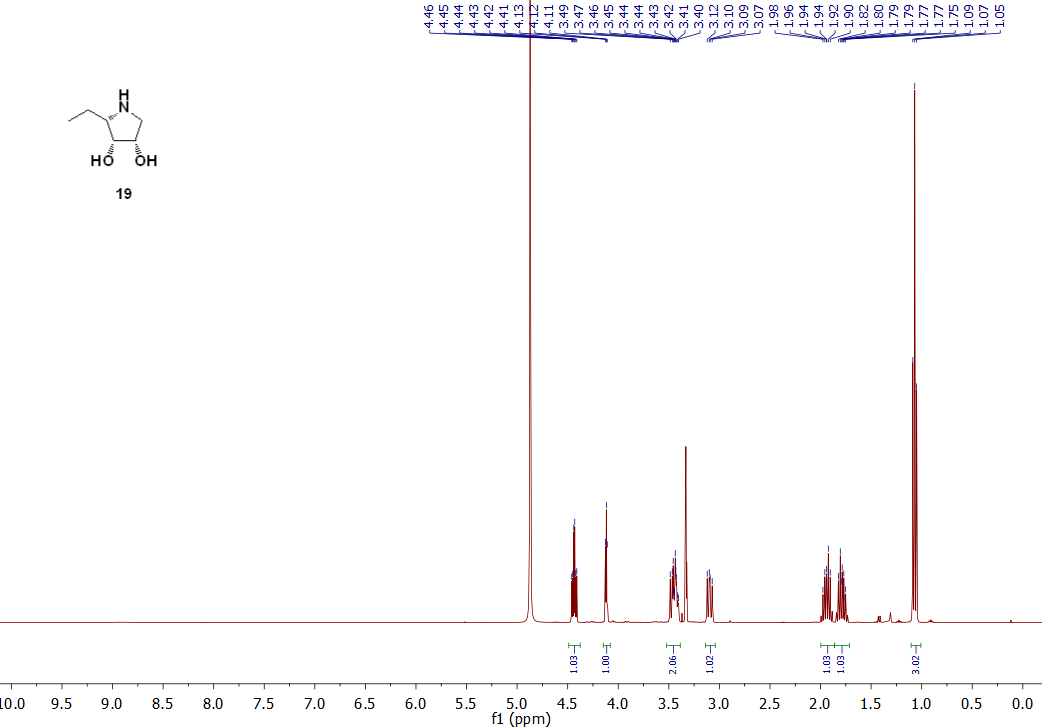


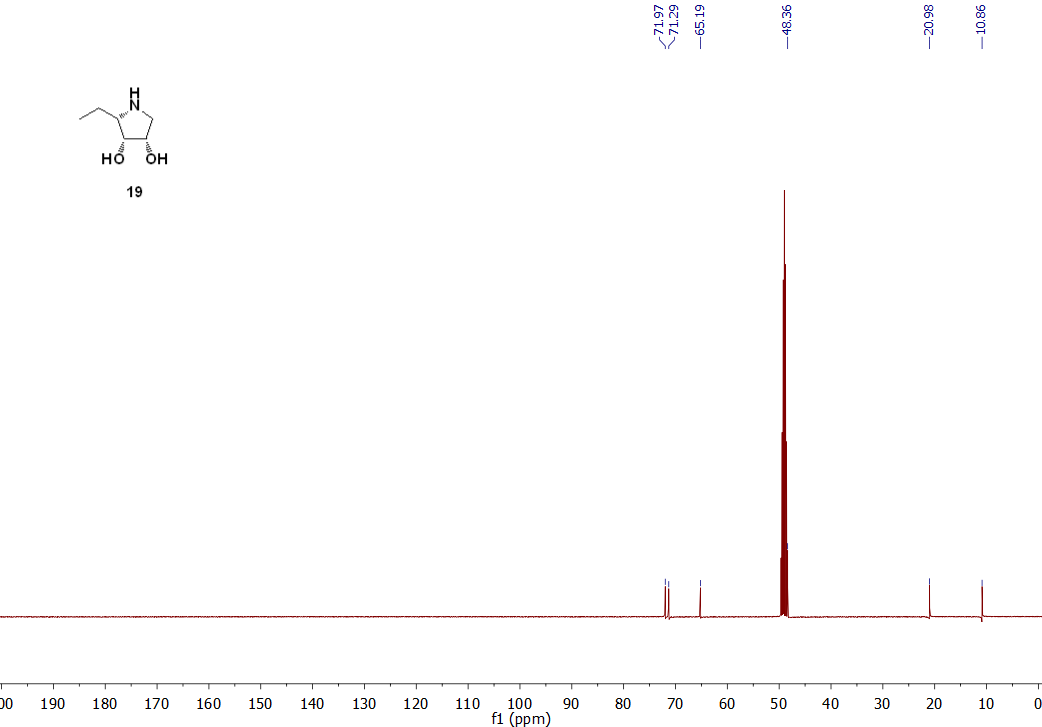


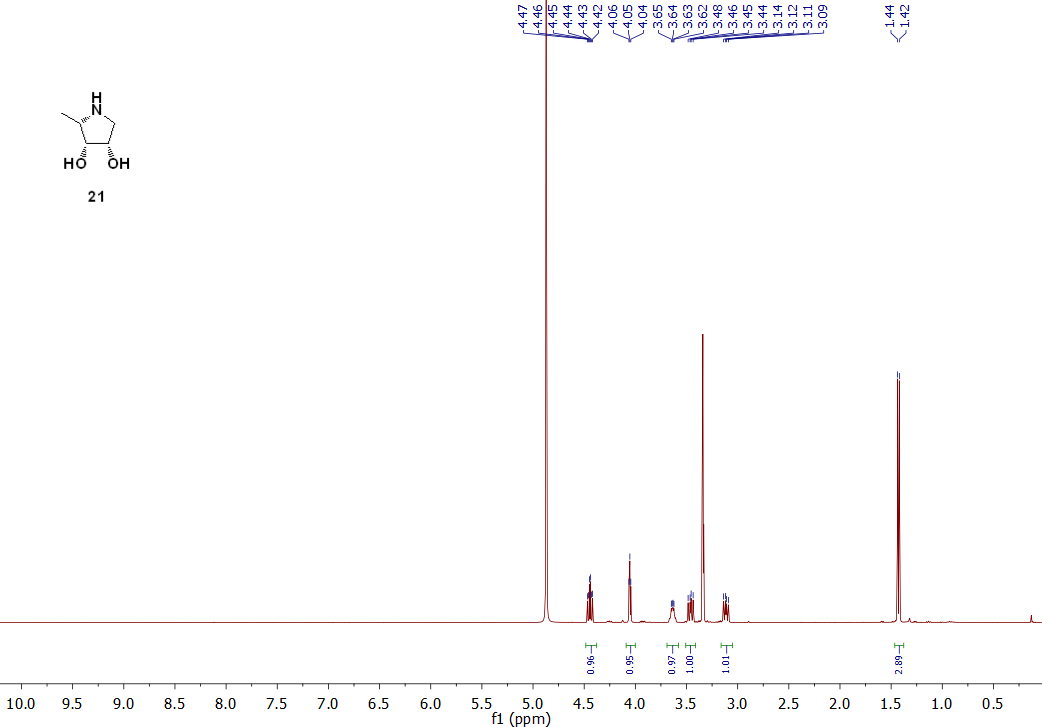


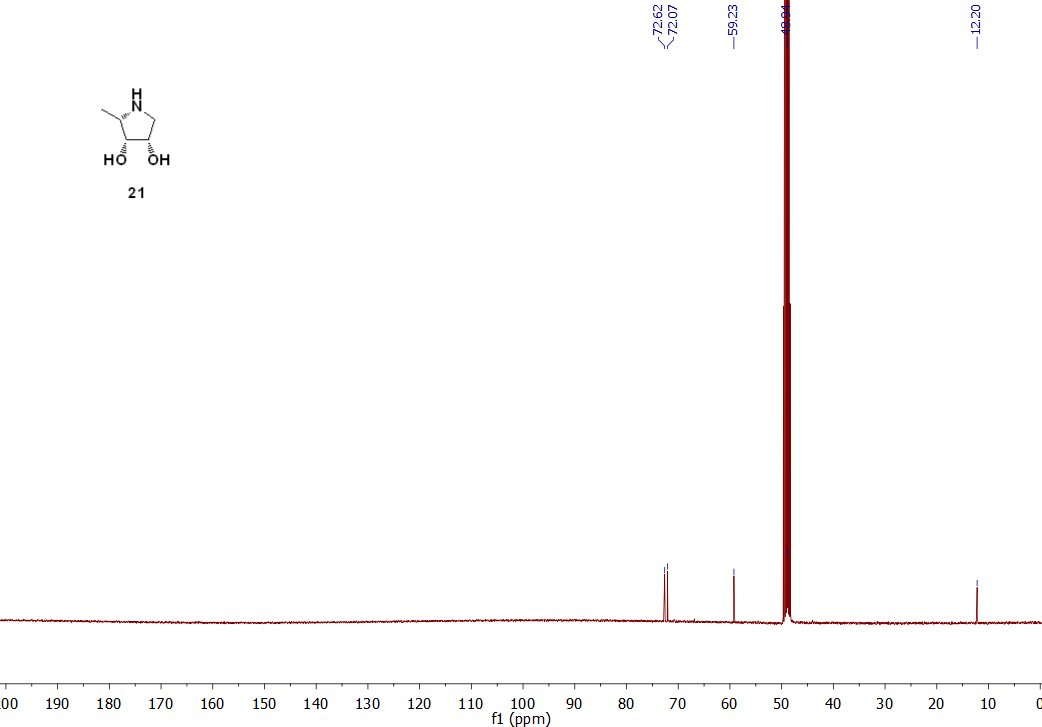

Supplement: Supporting information [file mmc1.docx]
